# Manually curated transcriptomics data collection for toxicogenomic assessment of engineered nanomaterials

**DOI:** 10.1038/s41597-021-00808-y

**Published:** 2021-02-08

**Authors:** Laura Aliisa Saarimäki, Antonio Federico, Iseult Lynch, Anastasios G. Papadiamantis, Andreas Tsoumanis, Georgia Melagraki, Antreas Afantitis, Angela Serra, Dario Greco

**Affiliations:** 1 Faculty of Medicine and Health Technology, Tampere University, Tampere, Finland; 2 BioMediTech Institute, Tampere University, Tampere, Finland; 3 School of Geography, Earth and Environmental Sciences, University of Birmingham, Edgbaston, B15 2TT Birmingham United Kingdom; 4 NovaMechanics Ltd, P.O Box 26014 1666, Nicosia, Cyprus; 5 Institute of Biotechnology, University of Helsinki, Helsinki, Finland; 6 Finnish Centre for Alternative Methods (FICAM), Faculty of Medicine and Heath Technology, Tampere University, Tampere, Finland

**Keywords:** Toxicology, Nanoparticles, Transcriptomics

## Abstract

Toxicogenomics (TGx) approaches are increasingly applied to gain insight into the possible toxicity mechanisms of engineered nanomaterials (ENMs). Omics data can be valuable to elucidate the mechanism of action of chemicals and to develop predictive models in toxicology. While vast amounts of transcriptomics data from ENM exposures have already been accumulated, a unified, easily accessible and reusable collection of transcriptomics data for ENMs is currently lacking. In an attempt to improve the FAIRness of already existing transcriptomics data for ENMs, we curated a collection of homogenized transcriptomics data from human, mouse and rat ENM exposures *in vitro* and *in vivo* including the physicochemical characteristics of the ENMs used in each study.

## Abstract (summary)

**Table:** 

Measurement(s)	microarray data • transcriptome • RNA • Toxicogenomics
Technology Type(s)	digital curation
Factor Type(s)	exposure to engineered nanomaterials
Sample Characteristic - Organism	Homo sapiens • Mus musculus • Rattus

Machine-accessible metadata file describing the reported data: 10.6084/m9.figshare.13154381

## Background & Summary

Engineered nanomaterials (ENMs) are an emerging class of chemicals with great technological and societal impact. Their unique physicochemical properties have already inspired multitudes of applications, ranging from medicine to industry and consumer products. While these unique properties make ENMs attractive for endless applications, they can also be responsible for potentially harmful effects on human health and the environment. ENMs can be synthesized in various sizes, shapes and chemistries with the smallest differences in the composition leading to novel properties and effects that need to be considered. Rigorous risk assessment is needed to ensure the safety of ENMs. Toxicogenomics (TGx) has emerged as a complementary approach to traditional toxicology with the potential to facilitate faster and cheaper hazard assessment of ENMs^1,2^. The large-scale profiling of exposure-induced molecular alterations sets the stage for mechanistic toxicology and expedites the development of predictive models. Furthermore, the application of TGx data to nanosafety can provide novel possibilities of grouping and classifying ENMs based on the similarity of molecular alterations in biological systems and furthermore can help to derive biomarkers to identify nano-specific signatures.

**Fig. 1:**
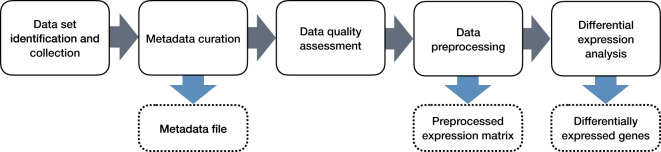
The workflow applied to compile the data collection. Solid-lined boxes represent the steps applied while the output is marked with a dashed line.

Transcriptomics technologies are the frontline of TGx. Vast amounts of transcriptomics data for multiple ENMs have already been generated offering a valuable resource for future studies and applications. However, the data are scattered across public repositories, and their FAIRness is currently hampered by their heterogeneous nature and lack of standardization in the preprocessing and analysis. The FAIR principles for scientific data were defined in 2016 and have since been the guide for more Findable, Accessible, Interoperable, and Reusable data^3^. The FAIRness of ENM-relevant databases, including ArrayExpress, the Gene Expression Omnibus (GEO), eNanoMapper and NanoCommons have recently been evaluated, and while the six datasets extracted from these met the majority of the criteria defined by the FAIR maturity indicators, areas identified for improvement included the use of standard schema for metadata and the presence of specific attributes in registries of repositories that would increase the FAIRness of datasets^4^. In order to unleash the full potential of already existing transcriptomics data on ENM exposures, which are lacking the metadata related to the exposure conditions and ENM characteristics, we created a unified collection of 101 manually curated and preprocessed data sets, covering a range of ENMs, organisms, and exposure setups, using the approach represented in Fig. 1.

The overarching aim of this study was to manually curate a comprehensive collection of transcriptomics data in the field of nanosafety, thereby increasing the degree of FAIRness of the original data sets. In particular, our collection is characterized by a higher degree of FAIRness as compared to the individual original data sets composing it.

## Methods

### Data set identification and collection

The first step in compiling the collection was to identify relevant data sets across public repositories. The search was limited to human, mouse, and rat data. We queried the Gene Expression Omnibus (GEO) and ArrayExpress databases with the following search terms: “engineered nanomaterial”, “nanomaterial” and “nanoparticle”. The initial collection yielded 124 unique entries, which went through manual assessment. Raw, non-normalized data for each microarray-based entry was downloaded from the series entry page, while for RNA-Seq data sets raw sequencing data in .fastq format were retrieved from the European Nucleotide Archive (ENA) (https://www.ebi.ac.uk/ena/browser/home).

### Metada**t**a curation

Next, supporting information (metadata) for each entry in the initial collection was downloaded and manually curated on R (version 3.5.2). Metadata gives context to the data by mapping each sample to biological variables, such as dose and time point, as well as technical variables crucial for the preprocessing of the data.

Metadata were obtained from the sample records of GEO entries by using the function *getGEO* from the R package GEOquery^5^. For data sets available only on ArrayExpress, the sample information for each entry was downloaded. These data were then manually curated to produce a homogenized file for each data set consisting of the following variables: *GSE* (a unique identifier for each data set)*, GSM* (sample id), *treatment* (exposure; *i.e*. ENM or control)*, group* (experimental group; combination of a unique exposure, dose, and time point)*, organism, biological system, dose, dose unit, time point, time point unit, slide, array, dye* and platform. Although some of these variables are not relevant for RNA-Seq data, all the columns were included for all the data to ensure convenient data usability. The nomenclature was unified to an extent that could be reached based on the information provided in the original metadata. Each sample was then mapped to its corresponding raw data file (column *filenames*) or annotated later to the fastq-files based on the sample names (GSM). If one or more predefined technical variables were missing, the column was left empty (NA). However, if biological variables were missing or ambiguous, the data set was discarded. Lastly, for entries containing human primary cells, the donor was further included in the metadata as an additional column *donor*.

### ENM physicochemical characteristics curation

The majority of the datasets were associated with a published article describing the study and including some details of the materials used and their physico-chemical characteristics. In some cases, the information provided was the nominal size information from the ENM manufacturer, while others provided more detailed characterization of the ENM in the exposure medium. Newer studies tended to provide more detailed characterization information than older ones, as the community knowledge regarding minimum characterization needs and properties influencing ENM toxicity increased^6,7^. Several of the studies utilized ENMs already used in previous studies and referred to the characterization provided in those earlier studies, in which case the information was manually extracted from the earlier papers. The curated information for the ENMs includes information on the supplier (including batch and lot information where available), the purity / impurities, the nominal size and surface area, as well as characterization data such as the core particle size (shape) as determined by Transmission Electron Microscopy (TEM) size, the hydrodynamic size and zeta potential (surface charge) in water and/or the exposure medium determined by Dynamic Light Scattering (DLS), information on the presence of endotoxin contamination (where provided) and a link to the commercial providers material specification sheet where relevant. As many of the studies utilized several different ENMs, or several variants (e.g. sizes, capping agents, polymeric coatings etc.) each individual ENM within each study is described in a separate row of the ENM characteristics datasheet.

### Manual quality assessment

The quality of transcriptomics data is highly dependent on the experimental design^2^. Low number of replicates results in weak statistics, while transcriptomics technologies themselves are often prone to technical bias. In order to ensure the quality and usability of each individual data set, evaluation was carried out based on the availability of raw data and supporting information as well as technical aspects of the experimental setup. The experiment was considered inappropriate for the collection if the experimental groups consisted of less than three biological replicates or if the experimental design introduced an unmanageable batch effect. Such batch effects were commonly introduced by consistently labeling different experimental groups with separate dyes in a two-color microarray experiment (i.e. lack of dye swapping). Furthermore, data sets representing non-commercial/custom or marginally represented platforms, for instance microarrays specific for miRNA or lncRNA, were excluded. As a result, only commercial gene expression microarrays from Agilent, Affymetrix, and Illumina were included alongside Illumina RNA-Seq platforms. The manual quality assessment of the collection is further described in the section *Technical Validation*.

### Data preprocessing

Preprocessing of transcriptomics data must be performed prior to any further analysis. The current standard preprocessing pipeline for microarray data includes steps for sample quality checking, probe filtering, data normalization, batch effect assessment and correction as well as probe annotation^8^. Similarly, the state-of-the-art preprocessing of RNA-Seq data includes quality control, read alignment, read count extraction, filtering low counts, normalization, and batch effect assessment^8^. Here, each data set was preprocessed and analyzed individually. Data sets consisting of several cell lines or tissues were further separated by the biological system to better focus on the transcriptional differences between the exposures.

**Fig. 2:**
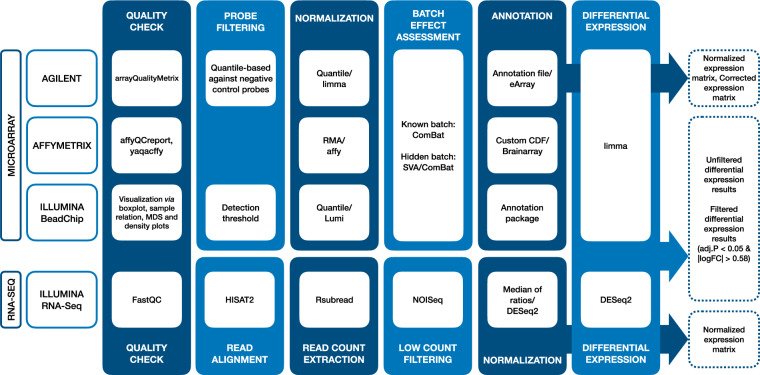
Preprocessing workflow applied to Agilent, Affymetrix, and Illumina microarrays and Illumina RNA-sequencing. Boxes with a blue background represent preprocessing steps and methods applied for each platform while boxes outlined with a dashed line represent the output obtained for each data set. The lack of a white box indicates that the step was not applied for the platform.

Preprocessing was performed in the R programming language (R version 3.5.2) following standard preprocessing pipelines suitable for each platform. For Agilent and Affymetrix microarrays, the preprocessing was implemented in the software eUTOPIA^9^. For Illumina BeadChips, a similar approach was applied following the suggested workflow of the R Bioconductor package lumi^10^. The preprocessing workflow applied to each platform is summarized in Fig. 2.

### Quality check

Omics data are prone to technical errors that can arise from sample handling as well as the intrinsic characteristics of the platforms^8^. For this, an important step prior to any manipulation of the data is the quality check (QC) that allows the assessment of the gene expression distributions across samples revealing outliers and poor-quality samples. We applied a platform specific QC on each data set to evaluate the quality of the samples as well as the prevalence of outliers in the data.

For Agilent microarrays, the R package arrayQualityMetrics^11^ was used, while the QC for Affymetrix was performed using the R packages affyQCreport^12^ and yaqcaffy^13^. Outliers were further assessed based on the visual representation in the form of density plots, bar plots, dendrograms, and multi-dimensional scaling (MDS) plots, which were also the primary method of outlier detection for Illumina arrays. Outliers were removed from subsequent preprocessing and analysis.

Quality checking of the RNA sequencing data was performed using FastQC v0.11.7 (https://www.bioinformatics.babraham.ac.uk/projects/fastqc/).

### Read alignment

RNA sequencing reads of mouse samples were aligned against the mouse reference genome assembly GRCm38, while sequencing reads of human samples were aligned against the human reference genome assembly GRCh38. The alignment was performed using the HISAT2 algorithm^14,15^ employing the genome indexes built for usage with HISAT2 (retrieved from https://ccb.jhu.edu/software/hisat2/manual.shtml). Sequencing file format conversions, such as.sam to.bam, sorting and extraction of uniquely mapped reads were performed using SAMtools (version 1.8-27-g0896262)^16^.

### Read counts extraction

Raw read counts for the RNA-Seq data were computed using the R package Rsubread (v2.2.3)^17^. The human Gencode version 35 annotation was applied for read counts extraction of human samples, while for mouse samples the mouse Gencode version M25 was employed. Both of the annotations were downloaded from https://www.gencodegenes.org.

### Low counts filtering

In order to filter out the transcripts with low expression levels in the samples of each RNA-Seq dataset, the proportion test was used as implemented in the Bioconductor NOISeq package (v2.31.0)^18^.

### Probe filtering

For microarray experiments, probe filtering is commonly applied to remove probes showing low variance in the intensity range similar to the background^8^. These low-intensity probes were removed prior to data normalization. For Agilent microarrays, filtering was based on estimating the robustness of the probe signal intensities against the background (negative control probes) and applying a quantile-based method for eliminating probes with low signals. Individual thresholds based on the data and the number of experimental groups and replicates were determined for Agilent. For Illumina gene expression microarrays, probe filtering was performed after normalization based on the detection p-values^10^ provided in the raw data. Only probes with a detection p-value < 0.01 in at least one sample were considered for further analysis.

### Normalization

Normalization of transcriptomics data is crucial for robust comparisons of gene expression. Here, the normalization of the expression signal distribution in the samples was performed on the log2 transformed signal intensities using the quantile normalization from the R package limma^19^ for Agilent, and the function *justRMA* from the package affy^20^ for Affymetrix microarrays, respectively. For Illumina microarrays, quantile normalization was performed with the function *lumiN* from the lumi R package^10^, while for Illumina RNA-Seq data, normalization was performed using the Bioconductor DESeq. 2 package^21^. In detail, the filtered raw counts underwent normalization by median of ratios method implemented in the package (for details see DESeq. 2 documentation).

### Batch effect assessment and correction

Microarray experiments are susceptible to technical variation arising from the experimental setup, sample preparation, and the equipment, for example. This type of variation can lead to decreased quality and incorrect results. Thus, reducing the variation associated with technical variables (batch effect), while maintaining biological variation, improves the robustness of the results. Here, batch effects were evaluated by inspecting the results of principal component analysis, hierarchical clustering and multi-dimensional scaling^9^. Technical variation arising from unknown batches were evaluated with the function *sva* from the R package sva^22^. If variation associated to known technical variables or any of the surrogate variables was observed, its correlation with biological variables of interest was assessed via a confounding plot^23^. Batches that were not confounded with any of the variables of interest were corrected using the *ComBat*^24^ function from the R package sva^22^.

### Probe annotation

Lastly, it is meaningful to map the probes to genes. For Agilent, the latest version of the annotation file for the specific microarray design was downloaded from the Agilent eArray website (https://earray.chem.agilent.com/earray/, 2020), and the probes were mapped to the Ensembl transcript IDs^25^. For Affymetrix gene expression arrays, the latest available alternative CDF files with Ensembl gene ID mappings were downloaded from Brainarray (http://brainarray.mbni.med.umich.edu/Brainarray/Database/CustomCDF/CDF_download.asp, 2020), while for Illumina BeadChips, the platform specific R annotation packages (illuminaHumanv3.db^26^, illuminaHumanv4.db^27^, illuminaRatv1.db^28^ or illuminaMousev2.db^29^) were used.

Multiple probes mapped onto the same gene ID were summarized by their median values. Agilent probes that were initially annotated to Ensembl transcripts were further mapped to the corresponding Ensembl gene IDs. If multiple transcripts were mapped to the same gene, the one with the highest absolute score, as calculated by the *-log(p-value) x log*_*2*_*(fold change)* for each exposure *vs*. control pairwise comparison, was selected.

### Differential expression analysis

Transcriptomics analysis aims at identifying gene expression differences between biological conditions. Here, we performed a differential expression analysis on each microarray data set using the R package limma^19^. Comparisons were made between each specific experimental group consisting of a single exposure, dose, and time point and its corresponding control samples. Batch corrected variables were included as covariates of the linear model. In case the biological material was obtained from human donors, the donor was included as a covariate for the analysis. For RNA-Seq based data sets similar comparisons were made using the Bioconductor DESeq. 2 package^21^.

As a result of the differential expression analysis, we provide full lists of genes with their specific fold changes and statistics as well as the results filtered to only contain significantly differentially expressed genes with the threshold of |logFC| > 0.58 and Benjamini & Hochberg adjusted p-value < 0.05. Due to the implementation of DESeq. 2 independent filtering (for details see DESeq. 2 documentation), we also computed the adjusted p-values for RNA-Seq data externally from DESeq. 2 to obtain the full list of adjusted p-values with no missing values. These values are included in the unfiltered result files of the differential expression analysis under the column “adj.P.Val.no.ind.filt”.

### FAIRness optimization

To further assist accessibility, interoperability and reusability, the data sets have been curated, imported and made publicly available from the NanoPharos database (https://db.nanopharos.eu/), which has been developed under the Horizon 2020 (H2020) NanoSolveIT^30^ (https://www.nanosolveit.eu) and NanoCommons projects (https://nanocommons.eu/). The NanoPharos database has been primarily developed to include computationally derived data based on simulations for ENMs at different levels of accuracy. The database was then further extended to include ENM characterization data and biological effects. With the inclusion of omics data, the NanoPharos database is now covering, in a ready for modelling format, the full spectrum of data needed to initiate a computational workflow for in silico exploitation of the data. The data set was checked for inconsistencies in the data structure and harmonized where needed. The ENM physico-chemical characterization data have been enriched, where applicable, with molecular (*e.g*. atomic/ionic radii, electronegativity, energy band gap) and structural (*e.g*. crystallographic space group, unit cell dimensions and angles). Each ENM has been linked to the respective transcriptomics data set to facilitate querying and user study. The datasets can be queried and grouped, among others, based on the ENM core material, ENM batch, exposure time and dose, biological information, experiment type, analysis platform etc. (Supplementary File 1).

The NanoPharos database has been designed under the FAIR data principles^3^ to offer users with high-quality, ready-for-modelling data sets, while allowing further development, adaptation and expansion. The FAIR data principles are meant to help database managers to improve data accessibility and reusability from the wider community in a way resembling Library Science^31^. To achieve this, data digitization in the NanoPharos database is being optimized to be machine readable to allow the seamless data comparison, transformation and, where possible, combination, providing the user with bigger and more complete data sets. On top of that, the NanoPharos database goes beyond the technical character of the FAIR data principles and is implementing the scientific FAIR data principles (SFAIR) as defined recently by Papadiamantis *et al*.^31^, providing users with the necessary scientific context and background information for them to be able to reuse the data with the highest possible confidence. Furthermore, NanoPharos is readily accessible via Representational State Transfer (REST) application programming interface (API) and is able to interact with external databases (*e.g*. NanoSolveIT Cloud) and modelling tools through API programmatic access. The available datasets can be accessed through: https://db.nanopharos.eu/Queries.

## Data Records

The data collection^32^ generated here is freely available on Zenodo at 10.5281/zenodo.4146981. The collection comprises 85 preprocessed microarray-based data sets totaling 506 unique ENM vs. control comparisons and 16 RNA-Seq based data sets representing 23 ENM vs. control comparisons. Additionally, 24 comparisons of non-nanoparticle compounds used as positive/negative controls in the original experiments are included for the microarray data sets and 7 additional compounds are included for the RNA-Seq data. All of the data sets and their descriptions are available in Online-only Table 1, while the physico-chemical characteristics of the tested ENMs are available in Online-only Table 2, respectively.

In order to facilitate the selection of data suitable for different applications and modelling approaches, we classified the data into four categories based on the experimental design as follows:

I – Multiple doses, multiple time points.

II – Multiple doses, one time point.

III – One dose, multiple time points.

IV – One dose, one time point.

**Fig. 3:**
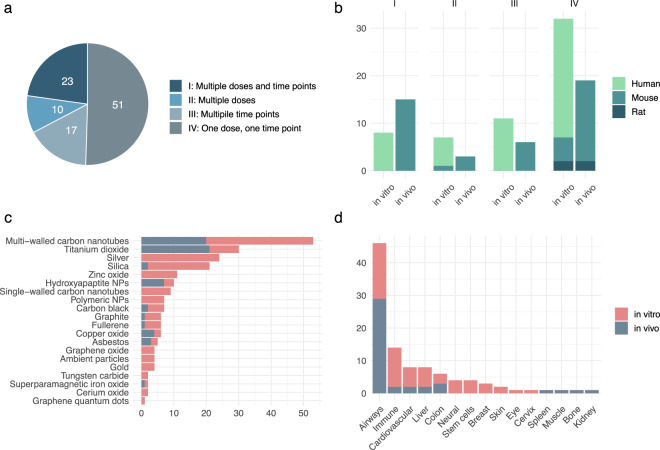
The data collection comprises of various experimental setups and exposures of multiple ENM compositions. (**a**) The total of 101 data sets were divided into four classes based on the experimental setup. The pie chart represents the distribution of data sets by class. (**b**) Bars representing the proportion of data sets in each organism divided by the four classes. In vivo and in vitro exposures are separated. (**c**) Horizontal bars represent the number of data sets with the specific ENM core material or material type. Grey bars represent in vivo exposures and pink bars in vitro exposures, respectively. (**d**) Bars represent the biological system used in the experiment. In vitro exposures are represented by pink bars and in vivo exposures by grey bars.

The proportion of each data class in the collection is visualized in Fig. 3a. Each class contains data obtained both *in vivo* and *in vitro* with at least two organisms represented (Fig. 3b). The collection covers a range of ENM compositions, as well as variants in size, shape, surface capping/coating etc. within a specific composition, in multiple biological systems in these organisms (Fig. 3c,d).

### Files available for each data set

Each data set contains a homogenized metadata file, normalized and batch corrected expression matrices as well as complete and filtered results of the differential expression analysis (Table 1).

**Table 1:** Files provided for each entry in the collection.

Output file	File extension	Description
**Metadata**	txt	Sample information containing the following columns: *GSE, GSM*, *treatment, group, organism, biological_system, dose, dose_unit, time_point, time_point_unit, slide, array, dye, platform, filenames*, (and *donor*).
**Normalized expression matrix**	txt	Ensembl IDs as row names, sample IDs (GSM) as column names. Values are log_2_-transformed and normalized signal intensities resulting from the preprocessing for microarrays, and normalized read counts for RNA-Seq data, respectively.
**Corrected expression matrix**	txt	Ensembl IDs as row names, sample IDs (GSM) as column names. Values are log_2_-transformed, normalized, and batch corrected signal intensities for microarrays. Only included for microarray-based entries for which applicable.
**Unfiltered differential expression results**	xlsx	Excel file containing a sheet for each comparison (experimental group *vs*. control group) in the data set entry. Each sheet is named “*group-control”* and contains the following columns: *LogFC, AveExpr, t-statistic, P.value, adj.P.Val, B-statistic, score* and *ID*, as specified in the output of the limma R package^16^ for microarrays. Columns available for RNA-Seq are *ID, baseMean, logFC, lfcSE, stat, P.Value, adj.P.Val* and *adj.P.Val.no.ind.filt*. Results contain all the genes in the platform after filtering and annotation.
**Filtered differential expression results**	xlsx	Excel file containing a sheet for each comparison with significantly differentially expressed genes with |logFC| > 0.58 and adj.P.Val < 0.05. Each sheet is named “*group-control”* and contains the following columns: *LogFC, AveExpr, t-statistic, P.value, adj.P.Val, B-statistic, score* and *ID*, as specified in the output of the limma R package^16^ for microarrays. Columns available for RNA-Seq are *ID, baseMean, logFC, lfcSE, stat, P.Value* and *adj.P.Val*. Only included for entries for which significantly altered genes were found.

## Technical Validation

The quality of transcriptomics data is a product of careful design of the experiment, technical execution as well as reporting of the data. The results of each downstream analysis substantially rely on the quality of the data. For this, we ensured that the collection contains high-quality data sets and defined a selection of criteria for data sets to be included:

- Three or more biological replicates are included for statistical robustness
- Microarray platform is a commercial gene expression microarray produced by Agilent, Affymetrix or Illumina
- The labelling of 2-color microarrays has been done considering dye swapping
- Non-normalized raw data is available
- Supporting information reports all variables required for preprocessing
- Untreated control samples are included

Each entry was evaluated based on the criteria, and either removed from the collection or selected for further preprocessing and analysis. The number of entries discarded for each of the listed reasons is represented in Table 2. Out of the 124 original entries 84 passed the quality assessment and were further divided into a total of 101 data sets (85 microarray and 16 RNA-Seq) based on the biological systems as specified in Data preprocessing.

**Table 2:** Reasons for discarding data during the manual quality assessment.

Reason to discard	Number of entries
Lack of replicates	26
Non-commercial or marginally represented platform	5
Two-color setup with no dye swapping	4
No raw data available	2
Incomplete metadata	2
Lack of control samples	1
Total entries discarded	40

## Usage Notes

Here we provide the biggest homogenized collection of transcriptomics data sets in the field of nanosafety supplemented with metadata and ENM physico-chemical characteristics. The collection offers a valuable source for multiple analysis and modeling approaches^33^. For instance, the mechanism of action of each ENM can be characterized by investigating the provided lists of differentially expressed genes, and may be linked to specific physico-chemical characteristics such as size, surface capping or coating which can guide redesign of ENMs that are safer and may support grouping into sets of nanoforms in accordance with REACH regulation (https://echa.europa.eu/documents/10162/13655/how_to_register_nano_en.pdf/f8c046ec-f60b-4349-492b-e915fd9e3ca0), for example. Moreover, pathway enrichment analysis can be performed to annotate these genes onto biological functions^34^. ENMs can be further compared and grouped based on the similarities between their molecular alteration profiles.

Due to the homogenized preprocessing and manual curation of the metadata, this collection is a relevant resource for identification of toxicity biomarkers. This can be addressed by using multiple feature selection approaches^35,36^ or more advanced data modelling techniques^37–39^. Biomarkers could also be detected by means of gene co-expression network analysis, under the assumption that central network genes play a key role in the adaptation to the exposure^40,41^.

The availability of data for multiple organisms or tissues can contribute to the development of more accurate adverse outcome pathways by linking ENM-specific molecular initiating events with cascades of relevant biological processes leading to an adverse outcome^42,43^. In addition, our data collection can be easily integrated with other transcriptomics data in the context of a read-across analysis to identify similarities in the molecular alterations induced by the ENMs with other phenotypic entities such as chemicals, drugs, and diseases^44^. Moreover, the data sets that we denoted as class I and II, where exposure at multiple doses are available, can be further analyzed to identify dose-dependent molecular alterations^45–48^.

Our manually curated transcriptomics data collection with supporting ENM descriptions will have a high impact on the nanosafety community and can aid the development of new methodologies for nanomaterial safety assessment^2,8,30,33,43^.

## Supplementary information

Supplementary File 1

## Data Availability

Preprocessing of the data was performed on R version 3.5.2. The preprocessing of Agilent and Affymetrix expression data was performed using eUTOPIA^9^, an R shiny software freely available on https://github.com/Greco-Lab/eUTOPIA. Custom scripts used for preprocessing of Illumina BeadChip and RNA sequencing data are available on GitHub on https://github.com/grecolab/Public_Nano.

## Online-only Tables

**Online-only Table 1:** Summary of data sets included in the collection.

ID	treatments	doses	time_points	n_samples	min_replicates	max_replicates	organism	biological_system	biological_system_specification	type	class	citation	doi	platform	technology	link_to_entry_page	files_provided
GSE112780	control, crocidolite, MWCNT	10 ug, 120 ug, 1 ug, 40 ug, 80 ug	12mo, 1mo, 6mo	139	5	9	Mus musculus	Lung	Whole organ	In vivo	I	Snyder-Talkington et al. 2016^49^	10.1080/15287394.2016.1159635	Affymetrix	DNA microarray	https://www.ncbi.nlm.nih.gov/geo/query/acc.cgi?acc=GSE112780	GSE112780_metadata.txt, GSE112780_Filtered_DEG.xlsx, GSE112780_Unfiltered_DEG.xlsx, Normalized_Expression_Matrix_GSE112780.txt
GSE29042	control, MWCNT	10 ug, 20 ug, 40 ug, 80 ug	1d, 28d, 56d, 7d	160	8	8	Mus musculus	Lung	Whole organ	In vivo	I	Dymacek & Guo, 2011^50^	10.1109/BIBM.2011.76	Agilent	DNA microarray	https://www.ncbi.nlm.nih.gov/geo/query/acc.cgi?acc=GSE29042	GSE29042_metadata.txt, Corrected_Expression_Matrix_GSE29042.txt, GSE29042_Filtered_DEG.xlsx, GSE29042_Unfiltered_DEG.xlsx, Normalized_Expression_Matrix_GSE29042.txt
GSE35193	Carbon black (Printex90), control	162 ug, 18 ug, 54 ug	1d, 28d, 3d	67	5	6	Mus musculus	Lung	Whole organ	In vivo	I	Bourdon et al. 2012^51^	10.1093/toxsci/kfs119	Agilent	DNA microarray	https://www.ncbi.nlm.nih.gov/geo/query/acc.cgi?acc=GSE35193	GSE35193_metadata.txt, Corrected_Expression_Matrix_GSE35193.txt, GSE35193_Filtered_DEG.xlsx, GSE35193_Unfiltered_DEG.xlsx, Normalized_Expression_Matrix_GSE35193.txt
GSE41041	control, nanoTiO2	162 ug, 18 ug, 54 ug	1d, 28d, 3d	65	5	6	Mus musculus	Lung	Whole organ	In vivo	I	Husain et al. 2013^52^	10.1016/j.taap.2013.03.018	Agilent	DNA microarray	https://www.ncbi.nlm.nih.gov/geo/query/acc.cgi?acc=GSE41041	GSE41041_metadata.txt, Corrected_Expression_Matrix_GSE41041.txt, GSE41041_Filtered_DEG.xlsx, GSE41041_Unfiltered_DEG.xlsx, Normalized_Expression_Matrix_GSE41041.txt
GSE42066	control, MWCNT, TiO2 nanobelt	100 ug/ml, 10 ug/ml	1h, 24h	30	3	3	Homo sapiens	Caco-2/HT29-MTX	Intestine epithelial cells	In vitro	I	Tilton et al. 2014^53^	10.3109/17435390.2013.803624	Affymetrix	DNA microarray	https://www.ncbi.nlm.nih.gov/geo/query/acc.cgi?acc=GSE42066	GSE42066_metadata.txt, GSE42066_Filtered_DEG.xlsx, GSE42066_Unfiltered_DEG.xlsx, Normalized_Expression_Matrix_GSE42066.txt
GSE42067	control, MWCNT, TiO2 nanobelt	100 ug/ml, 10 ug/ml	1h, 24h	30	3	3	Homo sapiens	SAE	Primary small airway epithelial cells	In vitro	I	Tilton et al. 2014^53^	10.3109/17435390.2013.803624	Affymetrix	DNA microarray	https://www.ncbi.nlm.nih.gov/geo/query/acc.cgi?acc=GSE42067	GSE42067_metadata.txt, Corrected_Expression_Matrix_GSE42067.txt, GSE42067_Filtered_DEG.xlsx, GSE42067_Unfiltered_DEG.xlsx, Normalized_Expression_Matrix_GSE42067.txt
GSE42068	control, MWCNT, TiO2 nanobelt	100 ug/ml, 10 ug/ml	1h, 24h	30	3	3	Homo sapiens	THP-1	Macrophage-like (PMA-differentiated)	In vitro	I	Tilton et al. 2014^53^	10.3109/17435390.2013.803624	Affymetrix	DNA microarray	https://www.ncbi.nlm.nih.gov/geo/query/acc.cgi?acc=GSE42068	GSE42068_metadata.txt, Corrected_Expression_Matrix_GSE42068.txt, GSE42068_Filtered_DEG.xlsx, GSE42068_Unfiltered_DEG.xlsx, Normalized_Expression_Matrix_GSE42068.txt
GSE51186	control, Eudragit RL, GSNO, GSNO-loaded Eudragit RL	200 ug/mL, 50 ug/mL, 50 uM	24h, 4h	34	3	5	Homo sapiens	THP-1	Monocyte	In vitro	I	Ronzani et al. 2014^54^	10.1093/jac/dku122	Agilent	DNA microarray	https://www.ncbi.nlm.nih.gov/geo/query/acc.cgi?acc=GSE51186	GSE51186_metadata.txt, GSE51186_Filtered_DEG.xlsx, GSE51186_Unfiltered_DEG.xlsx, Normalized_Expression_Matrix_GSE51186.txt
GSE55286	control, MWCNT (NM-401), MWCNT (NRCWE-26)	162 ug, 18 ug, 54 ug	1d, 28d, 3d	139	5	12	Mus musculus	Lung	Whole organ	In vivo	I	Poulsen et al. 2015^55^	10.1016/j.taap.2014.12.011	Agilent	DNA microarray	https://www.ncbi.nlm.nih.gov/geo/query/acc.cgi?acc=GSE55286	GSE55286_metadata.txt, Corrected_Expression_Matrix_GSE55286.txt, GSE55286_Filtered_DEG.xlsx, GSE55286_Unfiltered_DEG.xlsx, Normalized_Expression_Matrix_GSE55286.txt
GSE55349	Au40, Au5, control, control_Au40, control_Au5	100 uM, 300 uM	24h, 72h	42	3	3	Homo sapiens	Caco-2	Intestine epithelial cells	In vitro	I	Bajak et al. 2015^56^	10.1016/j.toxlet.2014.12.008	Agilent	DNA microarray	https://www.ncbi.nlm.nih.gov/geo/query/acc.cgi?acc=GSE55349	GSE55349_metadata.txt, Corrected_Expression_Matrix_GSE55349.txt, GSE55349_Filtered_DEG.xlsx, GSE55349_Unfiltered_DEG.xlsx, Normalized_Expression_Matrix_GSE55349.txt
GSE60797	control, Indoor-nanoTiO2 (sanding dust), Indoor-R (sanding dust)	162 ug, 486 ug, 54 ug	1d, 28d	70	4	6	Mus musculus	Lung	Whole organ	In vivo	I	Halappanavar et al. 2014^57^	10.1002/em.21936	Agilent	DNA microarray	https://www.ncbi.nlm.nih.gov/geo/query/acc.cgi?acc=GSE60797	GSE60797_metadata.txt, Corrected_Expression_Matrix_GSE60797.txt, GSE60797_Filtered_DEG.xlsx, GSE60797_Unfiltered_DEG.xlsx, Normalized_Expression_Matrix_GSE60797.txt
GSE60798	control, nanoTiO2 (NRCWE-001), nanoTiO2 (NRCWE-002)	162 ug, 18 ug, 54 ug	1d, 28d	69	4	5	Mus musculus	Lung	Whole organ	In vivo	I	Halappanavar et al. 2014^57^	10.1002/em.21936	Agilent	DNA microarray	https://www.ncbi.nlm.nih.gov/geo/query/acc.cgi?acc=GSE60798	GSE60798_metadata.txt, Corrected_Expression_Matrix_GSE60798.txt, GSE60798_Filtered_DEG.xlsx, GSE60798_Unfiltered_DEG.xlsx, Normalized_Expression_Matrix_GSE60798.txt
GSE60799	control, nanoTiO2 (NRCWE-025), nanoTiO2 (NRCWE-030)	162 ug, 18 ug, 54 ug	1d, 28d	69	4	5	Mus musculus	Lung	Whole organ	In vivo	I	Halappanavar et al. 2014^57^	10.1002/em.21936	Agilent	DNA microarray	https://www.ncbi.nlm.nih.gov/geo/query/acc.cgi?acc=GSE60799	GSE60799_metadata.txt, Corrected_Expression_Matrix_GSE60799.txt, GSE60799_Filtered_DEG.xlsx, GSE60799_Unfiltered_DEG.xlsx, Normalized_Expression_Matrix_GSE60799.txt
GSE60800	control, nanoTiO2 (NRCWE-032), nanoTiO2 (NRCWE-033)	162 ug, 18 ug, 54 ug	1d, 28d	70	4	6	Mus musculus	Lung	Whole organ	In vivo	I	Halappanavar et al. 2014^57^	10.1002/em.21936	Agilent	DNA microarray	https://www.ncbi.nlm.nih.gov/geo/query/acc.cgi?acc=GSE60800	GSE60800_metadata.txt, Corrected_Expression_Matrix_GSE60800.txt, GSE60800_Filtered_DEG.xlsx, GSE60800_Unfiltered_DEG.xlsx, Normalized_Expression_Matrix_GSE60800.txt
GSE63552	control, crocidolite, glass wool, MWCNT (Mitsui7)	0.25 ug/cm2, 2 ug/cm2	12h, 1h, 24h, 48h, 4h, 6h	123	2	3	Homo sapiens	BEAS-2B	Bronchial epithelial cells	In vitro	I	Nymark et al. 2015^58^	10.3109/17435390.2015.1017022	Affymetrix	DNA microarray	https://www.ncbi.nlm.nih.gov/geo/query/acc.cgi?acc=GSE63552	GSE63552_metadata.txt, GSE63552_Filtered_DEG.xlsx, GSE63552_Unfiltered_DEG.xlsx, Normalized_Expression_Matrix_GSE63552.txt
GSE63806	control, nanoSiO2	0.1 ug/cm2, 1.5 ug/cm2, 1 ug/cm2, 3 ug/cm2, 6 ug/cm2	24h, 72h	38	3	11	Homo sapiens	A549	Alveolar basal epithelial cells	In vitro	I	Pisani et al. 2015^59^	10.1186/s12864-015-1521-5	Agilent	DNA microarray	https://www.ncbi.nlm.nih.gov/geo/query/acc.cgi?acc=GSE63806	GSE63806_metadata.txt, Corrected_Expression_Matrix_GSE63806.txt, GSE63806_Filtered_DEG.xlsx, GSE63806_Unfiltered_DEG.xlsx, Normalized_Expression_Matrix_GSE63806.txt
GSE81564	control, nanoTiO2 (anatase)	162 ug, 486 ug, 54 ug	1d, 28d	37	4	5	Mus musculus	Lung	Whole organ	In vivo	I	Rahman et al. 2017^60^	10.1093/mutage/gew048	Agilent	DNA microarray	https://www.ncbi.nlm.nih.gov/geo/query/acc.cgi?acc=GSE81564	GSE81564_metadata.txt, Corrected_Expression_Matrix_GSE81564.txt, GSE81564_Filtered_DEG.xlsx, GSE81564_Unfiltered_DEG.xlsx, Normalized_Expression_Matrix_GSE81564.txt
GSE81565	control, nanoTiO2 (rutile hydrophilic)	162 ug, 486 ug, 54 ug	1d, 28d	39	4	5	Mus musculus	Lung	Whole organ	In vivo	I	Rahman et al. 2017^60^	10.1093/mutage/gew048	Agilent	DNA microarray	https://www.ncbi.nlm.nih.gov/geo/query/acc.cgi?acc=GSE81565	GSE81565_metadata.txt, Corrected_Expression_Matrix_GSE81565.txt, GSE81565_Filtered_DEG.xlsx, GSE81565_Unfiltered_DEG.xlsx, Normalized_Expression_Matrix_GSE81565.txt
GSE81566	control, nanoTiO2 (rutile hydrophobic)	162 ug, 486 ug, 54 ug	1d, 28d	37	4	5	Mus musculus	Lung	Whole organ	In vivo	I	Rahman et al. 2017^60^	10.1093/mutage/gew048	Agilent	DNA microarray	https://www.ncbi.nlm.nih.gov/geo/query/acc.cgi?acc=GSE81566	GSE81566_metadata.txt, Corrected_Expression_Matrix_GSE81566.txt, GSE81566_Filtered_DEG.xlsx, GSE81566_Unfiltered_DEG.xlsx, Normalized_Expression_Matrix_GSE81566.txt
GSE81567	control, nanoTiO2 (rutile/anatase mix)	162 ug, 486 ug, 54 ug	1d, 28d	40	5	5	Mus musculus	Lung	Whole organ	In vivo	I	Rahman et al. 2017^60^	10.1093/mutage/gew048	Agilent	DNA microarray	https://www.ncbi.nlm.nih.gov/geo/query/acc.cgi?acc=GSE81567	GSE81567_metadata.txt, Corrected_Expression_Matrix_GSE81567.txt, GSE81567_Filtered_DEG.xlsx, GSE81567_Unfiltered_DEG.xlsx, Normalized_Expression_Matrix_GSE81567.txt
GSE81568	control, nanoTiO2 (anatase)	162 ug, 486 ug, 54 ug	1d, 28d	40	5	5	Mus musculus	Lung	Whole organ	In vivo	I	Rahman et al. 2017^60^	10.1093/mutage/gew048	Agilent	DNA microarray	https://www.ncbi.nlm.nih.gov/geo/query/acc.cgi?acc=GSE81568	GSE81568_metadata.txt, Corrected_Expression_Matrix_GSE81568.txt, GSE81568_Filtered_DEG.xlsx, GSE81568_Unfiltered_DEG.xlsx, Normalized_Expression_Matrix_GSE81568.txt
GSE81569	control, nanoTiO2 (anatase)	162 ug, 486 ug, 54 ug	1d, 28d	40	5	5	Mus musculus	Lung	Whole organ	In vivo	I	Rahman et al. 2017^60^	10.1093/mutage/gew048	Agilent	DNA microarray	https://www.ncbi.nlm.nih.gov/geo/query/acc.cgi?acc=GSE81569	GSE81569_metadata.txt, Corrected_Expression_Matrix_GSE81569.txt, GSE81569_Filtered_DEG.xlsx, GSE81569_Unfiltered_DEG.xlsx, Normalized_Expression_Matrix_GSE81569.txt
GSE98236	control, M-MP-Silica NP (DMPC), M-MP-Silica NP (PEG), M-MP-Silica NP (Pristine)	1.6 ug/cm2, 16 ug/cm2, 80 ug/cm2	24h, 48h	68	3	7	Homo sapiens	HepaRG	Hepatocyte-like	In vitro	I	Pisani et al. 2017^61^	10.1080/17435390.2017.1378749	Agilent	DNA microarray	https://www.ncbi.nlm.nih.gov/geo/query/acc.cgi?acc=GSE98236	GSE98236_metadata.txt, GSE98236_Filtered_DEG.xlsx, GSE98236_Unfiltered_DEG.xlsx, Normalized_Expression_Matrix_GSE98236.txt
GSE122197	control_OVA, control_PBS, CuO_COOH_OVA, CuO_COOH_PBS, CuO_NH3_OVA, CuO_NH3_PBS, CuO_OVA, CuO_PBS, CuO_PEG_OVA, CuO_PEG_PBS	10 ug/day/4d, 2.5 ug/day/4d, 40 ug/day/4d	24h post exposure	89	3	8	Mus musculus	Lung (OVA/Alum sensitized)	Whole organ	In vivo	II	Ilves et al. 2019^62^	10.1186/s12989-019-0309-1	Agilent	DNA microarray	https://www.ncbi.nlm.nih.gov/geo/query/acc.cgi?acc=GSE122197	GSE122197_metadata.txt, Corrected_Expression_Matrix_GSE122197.txt, GSE122197_Filtered_DEG.xlsx, GSE122197_Unfiltered_DEG.xlsx, Normalized_Expression_Matrix_GSE122197.txt
GSE127773	control, CuO, CuO_COOH	120 mg/m3, 128 mg/m3, 23 mg/m3, 32 mg/m3, 470 mg/m3, 495 mg/m3	24h	64	3	10	Homo sapiens	Bronchial epithelium	MucilAirTM	In vitro	II	Kooter et al. 2019^63^	10.1021/acsnano.9b01823	Agilent	DNA microarray	https://www.ncbi.nlm.nih.gov/geo/query/acc.cgi?acc=GSE127773	GSE127773_metadata.txt, Corrected_Expression_Matrix_GSE127773.txt, GSE127773_Filtered_DEG.xlsx, GSE127773_Unfiltered_DEG.xlsx, Normalized_Expression_Matrix_GSE127773.txt
GSE117056	CeO2_Alfa_Aesar, CeO2_Nano_Amor, control, TiO2_Acros, TiO2_Degussa, TiO2_Mknano	0.3 ug/ml, 30 ug/ml, 3 ug/ml	3d	120	4	15	Homo sapiens	HepG2	Hepatocyte-like	In vitro	II	Thai et al. 2016^64^	No publication	Illumina	DNA microarray	https://www.ncbi.nlm.nih.gov/geo/query/acc.cgi?acc=GSE117056	GSE117056_metadata.txt, Corrected_Expression_Matrix_GSE117056.txt, GSE117056_Filtered_DEG.xlsx, GSE117056_Unfiltered_DEG.xlsx, Normalized_Expression_Matrix_GSE117056.txt
GSE30200	control, Silica	100 ug/ml, 200 ug/ml, 400 ug/ml	6h	20	5	5	Homo sapiens	A549	Alveolar basal epithelial cells	In vitro	II	Sellamuthu et al. 2011^65^	10.3109/08958378.2011.625995	Illumina	DNA microarray	https://www.ncbi.nlm.nih.gov/geo/query/acc.cgi?acc=GSE30200	GSE30200_metadata.txt, Corrected_Expression_Matrix_GSE30200.txt, GSE30200_Filtered_DEG.xlsx, GSE30200_Unfiltered_DEG.xlsx, Normalized_Expression_Matrix_GSE30200.txt
GSE46998	control, MWCNT	162 ug, 18 ug, 54 ug	24h	22	5	6	Mus musculus	Lung	Whole organ	In vivo	II	Søs Poulsen et al. 2013^66^	10.1371/journal.pone.0080452	Agilent	DNA microarray	https://www.ncbi.nlm.nih.gov/geo/query/acc.cgi?acc=GSE46998	GSE46998_metadata.txt, Corrected_Expression_Matrix_GSE46998.txt, GSE46998_Filtered_DEG.xlsx, GSE46998_Unfiltered_DEG.xlsx, Normalized_Expression_Matrix_GSE46998.txt
GSE46999	control, MWCNT	100 ug, 12.5 ug, 25 ug	24h	22	5	6	Mus musculus	FE1	Lung epithelial cells	In vitro	II	Søs Poulsen et al. 2013^66^	10.1371/journal.pone.0080452	Agilent	DNA microarray	https://www.ncbi.nlm.nih.gov/geo/query/acc.cgi?acc=GSE46999	GSE46999_metadata.txt, Corrected_Expression_Matrix_GSE46999.txt, GSE46999_Filtered_DEG.xlsx, GSE46999_Unfiltered_DEG.xlsx, Normalized_Expression_Matrix_GSE46999.txt
GSE62253	AgNO3, control, NanoAg	0.5 ug/ml, 2.5 ug/ml, 25 ug/ml	24h	12	3	3	Homo sapiens	Caco-2	Intestine epithelial cells	In vitro	II	Böhmert et al. 2015^67^	10.3109/17435390.2014.980760	Affymetrix	DNA microarray	https://www.ncbi.nlm.nih.gov/geo/query/acc.cgi?acc=GSE62253	GSE62253_metadata.txt, GSE62253_Filtered_DEG.xlsx, GSE62253_Unfiltered_DEG.xlsx, Normalized_Expression_Matrix_GSE62253.txt
GSE62769	asbestos, control, Silica	1.5e+07 um2/cm2, 7.5e+07 um2/cm2	24h	12	3	3	Homo sapiens	Primary human bronchial epithelial cells	Primary human bronchial epithelial cells	In vitro	II	Perkins et al. 2015^68^	10.1093/hmg/ddu551	Affymetrix	DNA microarray	https://www.ncbi.nlm.nih.gov/geo/query/acc.cgi?acc=GSE62769	GSE62769_metadata.txt, Corrected_Expression_Matrix_GSE62769.txt, GSE62769_Filtered_DEG.xlsx, GSE62769_Unfiltered_DEG.xlsx, Normalized_Expression_Matrix_GSE62769.txt
GSE75429	control, MWCNT (Mitsui7), MWCNT (NM-401)	128 ug, 42.7 ug	56d	26	5	6	Mus musculus	Lung	Whole organ	In vivo	II	Rahman et al. 2017^69^	10.1016/j.mrgentox.2017.08.005	Agilent	DNA microarray	https://www.ncbi.nlm.nih.gov/geo/query/acc.cgi?acc=GSE75429	GSE75429_metadata.txt, Corrected_Expression_Matrix_GSE75429.txt, GSE75429_Filtered_DEG.xlsx, GSE75429_Unfiltered_DEG.xlsx, Normalized_Expression_Matrix_GSE75429.txt
GSE16727	CoCl2, control, WC, WC-Co	30 ug/ml, 33 ug/ml, 51 uM	3hours, 72hours	40	5	5	Homo sapiens	HaCaT	Keratinocytes	In vitro	III	Busch et al. 2010^70^	10.1186/1471-2164-11-65	Agilent	DNA microarray	https://www.ncbi.nlm.nih.gov/geo/query/acc.cgi?acc=GSE16727	GSE16727_metadata.txt, Corrected_Expression_Matrix_GSE16727.txt, GSE16727_Filtered_DEG.xlsx, GSE16727_Unfiltered_DEG.xlsx, Normalized_Expression_Matrix_GSE16727.txt
GSE39330_donor	control, ZnO	10 ug/ml	24h, 6h	32	3	7	Homo sapiens	HMDM, MDDC	Human monocyte derived macrophages, human monocyte derived dendritic cells	In vitro	III	Tuomela et al. 2013^71^	10.1371/journal.pone.0068415.	Affymetrix	DNA microarray	https://www.ncbi.nlm.nih.gov/geo/query/acc.cgi?acc=GSE39330	GSE39330_donor_metadata.txt, GSE39330_donor_Filtered_DEG.xlsx, GSE39330_donor_Unfiltered_DEG.xlsx, Normalized_Expression_Matrix_GSE39330_donor.txt
GSE39330_jurkat	control, diethylene_glycol_ZnO, folic_acid_ZnO, mandelic_acid_ZnO, mercaptopropyl_trimethoxysilane_ZnO, methoxyl_ZnO, ZnO	100 ug/ml, 10 ug/ml, 25 ug/ml, 50 ug/ml	24h, 6h	39	3	6	Homo sapiens	Jurkat	T lymphocyte	In vitro	III	Tuomela et al. 2013^71^	10.1371/journal.pone.0068415.	Affymetrix	DNA microarray	https://www.ncbi.nlm.nih.gov/geo/query/acc.cgi?acc=GSE39330	GSE39330_jurkat_metadata.txt, Corrected_Expression_Matrix_GSE39330_jurkat.txt, GSE39330_jurkat_Filtered_DEG.xlsx, GSE39330_jurkat_Unfiltered_DEG.xlsx, Normalized_Expression_Matrix_GSE39330_jurkat.txt
GSE43515	Carbon black, Carbon black_IL13, control, control_IL13, MWCNT, MWCNT_IL13	10 ug/cm2	24h, 48h, 4h	54	3	3	Homo sapiens	16lu	Lung fibroblast	In vitro	III	Martin et al. 2013^72^	No publication	Affymetrix	DNA microarray	https://www.ncbi.nlm.nih.gov/geo/query/acc.cgi?acc=GSE43515	GSE43515_metadata.txt, Corrected_Expression_Matrix_GSE43515.txt, GSE43515_Filtered_DEG.xlsx, GSE43515_Unfiltered_DEG.xlsx, Normalized_Expression_Matrix_GSE43515.txt
GSE45322	control, ZnO_HP1, ZnO_MAX, ZnO_Nanosun, ZnO_ZCOTE	25 ug/ml	2h, 6h	40	4	4	Homo sapiens	hONS	human olfactory neurosphere derived cells	In vitro	III	Osmond-McLeod et al. 2013^73^	10.1186/1743-8977-10-54	Affymetrix	DNA microarray	https://www.ncbi.nlm.nih.gov/geo/query/acc.cgi?acc=GSE45322	GSE45322_metadata.txt, Corrected_Expression_Matrix_GSE45322.txt, GSE45322_Filtered_DEG.xlsx, GSE45322_Unfiltered_DEG.xlsx, Normalized_Expression_Matrix_GSE45322.txt
GSE51417	control, SPIO	365 ug	0h, 168h, 24h, 48h, 6h, 96h	58	4	5	Mus musculus	Lung	Whole organ	In vivo	III	Teeguarden et al. 2014^74^	10.1186/s12989-014-0046-4	Affymetrix	DNA microarray	https://www.ncbi.nlm.nih.gov/geo/query/acc.cgi?acc=GSE51417	GSE51417_metadata.txt, Corrected_Expression_Matrix_GSE51417.txt, GSE51417_Filtered_DEG.xlsx, GSE51417_Unfiltered_DEG.xlsx, Normalized_Expression_Matrix_GSE51417.txt
GSE51421	control, PLGA	500 ug/ml	24h, 7d	12	3	3	Homo sapiens	CD34	Hematopoietic stem cells (CD34+ fraction of umbilical cord blood mononuclear cells)	In vitro	III	Aday et al. 2014^75^	10.1039/C4RA04041D	Agilent	DNA microarray	https://www.ncbi.nlm.nih.gov/geo/query/acc.cgi?acc=GSE51421	GSE51421_metadata.txt, GSE51421_Filtered_DEG.xlsx, GSE51421_Unfiltered_DEG.xlsx, Normalized_Expression_Matrix_GSE51421.txt
GSE53700	control, control_SM30, SiO2_AS30, SiO2_SM30	0.03 mg/ml	22h, 3h	18	3	3	Homo sapiens	A549	Alveolar basal epithelial cells	In vitro	III	Fede et al. 2014^76^	10.3390/ijerph110908867	Agilent	DNA microarray	https://www.ncbi.nlm.nih.gov/geo/query/acc.cgi?acc=GSE53700	GSE53700_metadata.txt, Corrected_Expression_Matrix_GSE53700.txt, GSE53700_Filtered_DEG.xlsx, GSE53700_Unfiltered_DEG.xlsx, Normalized_Expression_Matrix_GSE53700.txt
GSE56324	control, nanoTiO2	162 ug	1d, 28d	22	5	6	Mus musculus	heart	Whole organ	In vivo	III	Husain et al. 2015^77^	10.3109/17435390.2014.996192	Agilent	DNA microarray	https://www.ncbi.nlm.nih.gov/geo/query/acc.cgi?acc=GSE56324	GSE56324_metadata.txt, Corrected_Expression_Matrix_GSE56324.txt, GSE56324_Unfiltered_DEG.xlsx, Normalized_Expression_Matrix_GSE56324.txt
GSE56325	control, nanoTiO2	162 ug	1d, 28d	21	4	6	Mus musculus	liver	Whole organ	In vivo	III	Husain et al. 2015^77^	10.3109/17435390.2014.996192	Agilent	DNA microarray	https://www.ncbi.nlm.nih.gov/geo/query/acc.cgi?acc=GSE56325	GSE56325_metadata.txt, Corrected_Expression_Matrix_GSE56325.txt, GSE56325_Unfiltered_DEG.xlsx, Normalized_Expression_Matrix_GSE56325.txt
GSE61366	control_NCRWE-026, control_NM-401, MWCNT (NCRWE-026), MWCNT (NM-401)	162 ug	1d, 28d, 3d	60	5	5	Mus musculus	liver	Whole organ	In vivo	III	Poulsen et al. 2015^78^	10.1016/j.taap.2015.01.011	Agilent	DNA microarray	https://www.ncbi.nlm.nih.gov/geo/query/acc.cgi?acc=GSE61366	GSE61366_metadata.txt, Corrected_Expression_Matrix_GSE61366.txt, GSE61366_Filtered_DEG.xlsx, GSE61366_Unfiltered_DEG.xlsx, Normalized_Expression_Matrix_GSE61366.txt
GSE68036	Carbon black (Printex90), control	162 ug	14d, 1d, 2d, 3d, 3h, 42d, 4d, 5d	76	4	5	Mus musculus	Lung	Whole organ	In vivo	III	Husain et al. 2015^79^	10.1016/j.taap.2015.11.003	Agilent	DNA microarray	https://www.ncbi.nlm.nih.gov/geo/query/acc.cgi?acc=GSE68036	GSE68036_metadata.txt, Corrected_Expression_Matrix_GSE68036.txt, GSE68036_Filtered_DEG.xlsx, GSE68036_Unfiltered_DEG.xlsx, Normalized_Expression_Matrix_GSE68036.txt
GSE84982_Caco2	110nm_AgNPs, 20nm_AgNPs, 30nm_AgNPs, 60nm_AgNPs, AgNO3, control	1.5 ug/ml, 25 ug/ml	24h, 6h	36	3	3	Homo sapiens	Caco-2	Intestine epithelial cells	In vitro	III	van der Zande et al. 2016^80^	10.1080/17435390.2016.1225132	Illumina	DNA microarray	https://www.ncbi.nlm.nih.gov/geo/query/acc.cgi?acc=GSE84982	GSE84982_Caco2_metadata.txt, Corrected_Expression_Matrix_GSE84982_Caco2.txt, GSE84982_Caco2_Filtered_DEG.xlsx, GSE84982_Caco2_Unfiltered_DEG.xlsx, Normalized_Expression_Matrix_GSE84982_Caco2.txt
GSE84982_MCF7	110nm_AgNPs, 20nm_AgNPs, 30nm_AgNPs, 60nm_AgNPs, AgNO3, control	1.5 ug/ml, 25 ug/ml	24h, 6h	37	3	4	Homo sapiens	MCF-7	Mammary gland epitelial cells	In vitro	III	van der Zande et al. 2016^80^	10.1080/17435390.2016.1225132	Illumina	DNA microarray	https://www.ncbi.nlm.nih.gov/geo/query/acc.cgi?acc=GSE84982	GSE84982_MCF7_metadata.txt, Corrected_Expression_Matrix_GSE84982_MCF7.txt, GSE84982_MCF7_Filtered_DEG.xlsx, GSE84982_MCF7_Unfiltered_DEG.xlsx, Normalized_Expression_Matrix_GSE84982_MCF7.txt
GSE92899	control, Fullerene, Graphite, MWCNT (Baytube), MWCNT (SES), rCNT, tCNT	100 ug/ml	24h, 6h	47	3	6	Homo sapiens	THP-1	Macrophage-like (PMA-differentiated)	In vitro	III	Kinaret et al. 2017^81^	10.1021/acsnano.6b08650	Agilent	DNA microarray	https://www.ncbi.nlm.nih.gov/geo/query/acc.cgi?acc=GSE92899	GSE92899_metadata.txt, Corrected_Expression_Matrix_GSE92899.txt, GSE92899_Filtered_DEG.xlsx, GSE92899_Unfiltered_DEG.xlsx, Normalized_Expression_Matrix_GSE92899.txt
GSE88786	control, Fe3O4_TiO2_NP	20 ug/ml	2h, 4h	20	5	5	Homo sapiens	HeLa	Cervix, epithelial	In vitro	III	Lastra et al. 2019^82^	10.33218/prnano2(4).190802.1	Illumina	DNA microarray	https://www.ncbi.nlm.nih.gov/geo/query/acc.cgi?acc=GSE88786	GSE88786_metadata.txt, Corrected_Expression_Matrix_GSE88786.txt, GSE88786_Filtered_DEG.xlsx, GSE88786_Unfiltered_DEG.xlsx, Normalized_Expression_Matrix_GSE88786.txt
EMTAB6396_A549	Carbon black, control, Fullerene, Graphite nanofibers, MWCNT (Bayer), MWCNT (Cheaptubes), MWCNT (Mitsui), MWCNT (SES), MWCNT (Sigma), SWCNT (SES), SWCNT (Sigma)	10 ug/ml	48h	32	2	3	Homo sapiens	A549	Alveolar basal epithelial cells	In vitro	IV	Scala & Kinaret et al. 2018^83^	10.1016/j.impact.2018.05.003	Agilent	DNA microarray	https://www.ebi.ac.uk/arrayexpress/experiments/E-MTAB-6396/	EMTAB6396_A549_metadata.txt, Corrected_Expression_Matrix_EMTAB6396_A549.txt, EMTAB6396_A549_Filtered_DEG.xlsx, EMTAB6396_A549_Unfiltered_DEG.xlsx, Normalized_Expression_Matrix_EMTAB6396_A549.txt
EMTAB6396_Beas2B	Carbon black, control, Fullerene, Graphite nanofibers, MWCNT (Bayer), MWCNT (Cheaptubes), MWCNT (Mitsui), MWCNT (SES), MWCNT (Sigma), SWCNT (SES), SWCNT (Sigma)	10 ug/ml	48h	32	2	3	Homo sapiens	BEAS-2B	Bronchial epithelial cells	In vitro	IV	Scala & Kinaret et al. 2018^83^	10.1016/j.impact.2018.05.003	Agilent	DNA microarray	https://www.ebi.ac.uk/arrayexpress/experiments/E-MTAB-6396/	EMTAB6396_Beas2B_metadata.txt, Corrected_Expression_Matrix_EMTAB6396_Beas2B.txt, EMTAB6396_Beas2B_Filtered_DEG.xlsx, EMTAB6396_Beas2B_Unfiltered_DEG.xlsx, Normalized_Expression_Matrix_EMTAB6396_Beas2B.txt
EMTAB6396_MRC9	Carbon black, control, Fullerene, Graphite nanofibers, MWCNT (Bayer), MWCNT (Cheaptubes), MWCNT (Mitsui), MWCNT (SES), SWCNT (SES), MWCNT (Sigma), SWCNT (Sigma)	10 ug/ml	48h	32	2	3	Homo sapiens	MRC9	Lung fibroblast	In vitro	IV	Scala & Kinaret et al. 2018^83^	10.1016/j.impact.2018.05.003	Agilent	DNA microarray	https://www.ebi.ac.uk/arrayexpress/experiments/E-MTAB-6396/	EMTAB6396_MRC9_metadata.txt, Corrected_Expression_Matrix_EMTAB6396_MRC9.txt, EMTAB6396_MRC9_Filtered_DEG.xlsx, EMTAB6396_MRC9_Unfiltered_DEG.xlsx, Normalized_Expression_Matrix_EMTAB6396_MRC9.txt
EMTAB6396_THP1	Carbon black, control, Fullerene, Graphite nanofibers, MWCNT (Bayer), MWCNT (Cheaptubes), MWCNT (Mitsui), MWCNT (SES), MWCNT (Sigma), SWCNT (SES), SWCNT (Sigma)	10 ug/ml	48h	32	2	3	Homo sapiens	THP-1	Macrophage-like (PMA-differentiated)	In vitro	IV	Scala & Kinaret et al. 2018^83^	10.1016/j.impact.2018.05.003	Agilent	DNA microarray	https://www.ebi.ac.uk/arrayexpress/experiments/E-MTAB-6396/	EMTAB6396_THP1_metadata.txt, Corrected_Expression_Matrix_EMTAB6396_THP1.txt, EMTAB6396_THP1_Filtered_DEG.xlsx, EMTAB6396_THP1_Unfiltered_DEG.xlsx, Normalized_Expression_Matrix_EMTAB6396_THP1.txt
GSE100500	control, MWCNT	100 ug	60d	12	6	6	Mus musculus	Alveolar macrophage	Primary murine alveolar macrophage	In vivo	IV	Mohan et al. 2018^84^	10.1152/ajplung.00289.2017	Illumina	DNA microarray	https://www.ncbi.nlm.nih.gov/geo/query/acc.cgi?acc=GSE100500	GSE100500_metadata.txt, Corrected_Expression_Matrix_GSE100500.txt, GSE100500_Filtered_DEG.xlsx, GSE100500_Unfiltered_DEG.xlsx, Normalized_Expression_Matrix_GSE100500.txt
GSE103101	AgNPs_100nm, AgNPs_5nm, control	50 LD	6h	9	3	3	Homo sapiens	EA.hy926	Somatic cell hybrid, endothelial	In vitro	IV	Jang & Choi 2017^85^	No publication	Affymetrix	DNA microarray	https://www.ncbi.nlm.nih.gov/geo/query/acc.cgi?acc=GSE103101	GSE103101_metadata.txt, GSE103101_Filtered_DEG.xlsx, GSE103101_Unfiltered_DEG.xlsx, Normalized_Expression_Matrix_GSE103101.txt
GSE113088	control, control_saline, nanoSiO2	150 uM	0h, 4h	9	3	3	Homo sapiens	PBMC	Peripheral blood mononuclear cells	In vitro	IV	Vis et al. 2018^86^	10.1021/acsnano.8b03363	Affymetrix	DNA microarray	https://www.ncbi.nlm.nih.gov/geo/query/acc.cgi?acc=GSE113088	GSE113088_metadata.txt, GSE113088_Filtered_DEG.xlsx, GSE113088_Unfiltered_DEG.xlsx, Normalized_Expression_Matrix_GSE113088.txt
GSE14452	Ag2CO3, AgNPs, AgNPs_cysteine, control, PS	1.3 mg/l, 1 mg/l	24h	15	3	3	Homo sapiens	HepG2	Hepatocyte-like	In vitro	IV	Kawata et al. 2009^87^	10.1021/es900754q	Affymetrix	DNA microarray	https://www.ncbi.nlm.nih.gov/geo/query/acc.cgi?acc=GSE14452	GSE14452_metadata.txt, GSE14452_Filtered_DEG.xlsx, GSE14452_Unfiltered_DEG.xlsx, Normalized_Expression_Matrix_GSE14452.txt
GSE17676	flat_titanium (control), TiO2 nanotubes (100nm), TiO2 nanotubes (30nm)	NA	24h	12	4	4	Homo sapiens	HAEC	Primary human aortic endothelial cells	In vitro	IV	Peng et al. 2010^88^	10.1021/nl903043z	Agilent	DNA microarray	https://www.ncbi.nlm.nih.gov/geo/query/acc.cgi?acc=GSE17676	GSE17676_metadata.txt, Corrected_Expression_Matrix_GSE17676.txt, GSE17676_Filtered_DEG.xlsx, GSE17676_Unfiltered_DEG.xlsx, Normalized_Expression_Matrix_GSE17676.txt
GSE19487_liver	control, nanoTiO2	40 mg/m3	5d	15	7	8	Mus musculus	liver	Whole organ	In vivo	IV	Halappanavar et al. 2011^89^	10.1002/em.20639	Agilent	DNA microarray	https://www.ncbi.nlm.nih.gov/geo/query/acc.cgi?acc=GSE19487	GSE19487_liver_metadata.txt, Corrected_Expression_Matrix_GSE19487_liver.txt, GSE19487_liver_Unfiltered_DEG.xlsx, Normalized_Expression_Matrix_GSE19487_liver.txt
GSE19487_lung	control, nanoTiO2	40 mg/m3	5d	13	6	7	Mus musculus	Lung	Whole organ	In vivo	IV	Halappanavar et al. 2011^89^	10.1002/em.20639	Agilent	DNA microarray	https://www.ncbi.nlm.nih.gov/geo/query/acc.cgi?acc=GSE19487	GSE19487_lung_metadata.txt, GSE19487_lung_Filtered_DEG.xlsx, GSE19487_lung_Unfiltered_DEG.xlsx, Normalized_Expression_Matrix_GSE19487_lung.txt
GSE20692	Ag_ions, AgNPs, control	0.2 mg/l	24h	9	3	3	Homo sapiens	Jurkat	T lymphocyte	In vitro	IV	Eom et al. 2014^90^	10.1016/j.toxlet.2014.05.019.	Illumina	DNA microarray	https://www.ncbi.nlm.nih.gov/geo/query/acc.cgi?acc=GSE20692	GSE20692_metadata.txt, Corrected_Expression_Matrix_GSE20692.txt, GSE20692_Filtered_DEG.xlsx, GSE20692_Unfiltered_DEG.xlsx, Normalized_Expression_Matrix_GSE20692.txt
GSE27212	control_polyornithine, MWCNT	NA	8days	6	3	3	Rattus norvegicus	Spinal cord dissociated cultures	Dissociated spinal neurons	In vitro	IV	Fabbro et al. 2013^91^	10.1371/journal.pone.0073621	Affymetrix	DNA microarray	https://www.ncbi.nlm.nih.gov/geo/query/acc.cgi?acc=GSE27212	GSE27212_metadata.txt, GSE27212_Unfiltered_DEG.xlsx, Normalized_Expression_Matrix_GSE27212.txt
GSE29110	control, Silica	6.2 mg/m3	28week	6	3	3	Rattus norvegicus	Lung	Whole organ	In vivo	IV	Langley et al. 2011^92^	10.1080/15287394.2011.595669	Affymetrix	DNA microarray	https://www.ncbi.nlm.nih.gov/geo/query/acc.cgi?acc=GSE29110	GSE29110_metadata.txt, GSE29110_Filtered_DEG.xlsx, GSE29110_Unfiltered_DEG.xlsx, Normalized_Expression_Matrix_GSE29110.txt
GSE30178	control, Silica	15 mg/m3	0week	20	8	12	Rattus norvegicus	Lung	Whole organ	In vivo	IV	Sellamuthu et al. 2011^65^	10.3109/08958378.2011.625995	Illumina	DNA microarray	https://www.ncbi.nlm.nih.gov/geo/query/acc.cgi?acc=GSE30178	GSE30178_metadata.txt, Corrected_Expression_Matrix_GSE30178.txt, GSE30178_Filtered_DEG.xlsx, GSE30178_Unfiltered_DEG.xlsx, Normalized_Expression_Matrix_GSE30178.txt
GSE30180	control, Silica	800 ug/ml	6h	10	5	5	Homo sapiens	A549	Alveolar basal epithelial cells	In vitro	IV	Sellamuthu et al. 2011^65^	10.3109/08958378.2011.625995	Illumina	DNA microarray	https://www.ncbi.nlm.nih.gov/geo/query/acc.cgi?acc=GSE30180	GSE30180_metadata.txt, GSE30180_Filtered_DEG.xlsx, GSE30180_Unfiltered_DEG.xlsx, Normalized_Expression_Matrix_GSE30180.txt
GSE30213	control, Silica	50 ug/ml	6h	9	4	5	Homo sapiens	A549	Alveolar basal epithelial cells	In vitro	IV	Sellamuthu et al. 2011^65^	10.3109/08958378.2011.625995	Illumina	DNA microarray	https://www.ncbi.nlm.nih.gov/geo/query/acc.cgi?acc=GSE30213	GSE30213_metadata.txt, Corrected_Expression_Matrix_GSE30213.txt, GSE30213_Filtered_DEG.xlsx, GSE30213_Unfiltered_DEG.xlsx, Normalized_Expression_Matrix_GSE30213.txt
GSE30214	control, Silica	200 ug/ml	2h	8	3	5	Homo sapiens	A549	Alveolar basal epithelial cells	In vitro	IV	Sellamuthu et al. 2011^65^	10.3109/08958378.2011.625995	Illumina	DNA microarray	https://www.ncbi.nlm.nih.gov/geo/query/acc.cgi?acc=GSE30214	GSE30214_metadata.txt, Corrected_Expression_Matrix_GSE30214.txt, GSE30214_Filtered_DEG.xlsx, GSE30214_Unfiltered_DEG.xlsx, Normalized_Expression_Matrix_GSE30214.txt
GSE30215	control, Silica	200 ug/ml	24h	9	4	5	Homo sapiens	A549	Alveolar basal epithelial cells	In vitro	IV	Sellamuthu et al. 2011^65^	10.3109/08958378.2011.625995	Illumina	DNA microarray	https://www.ncbi.nlm.nih.gov/geo/query/acc.cgi?acc=GSE30215	GSE30215_metadata.txt, GSE30215_Filtered_DEG.xlsx, GSE30215_Unfiltered_DEG.xlsx, Normalized_Expression_Matrix_GSE30215.txt
GSE44294	control, LPS, SiO2, SiO2_LPS, SPIO, SPIO_LPS	10 ng/ml, 25 mg/ml	4h	18	3	3	Mus musculus	BMM	Bone-marrow derived macrophages	In vitro	IV	Kodali et al. 2013^93^	10.1021/nn402145t	Affymetrix	DNA microarray	https://www.ncbi.nlm.nih.gov/geo/query/acc.cgi?acc=GSE44294	GSE44294_metadata.txt, GSE44294_Filtered_DEG.xlsx, GSE44294_Unfiltered_DEG.xlsx, Normalized_Expression_Matrix_GSE44294.txt
GSE45598	control, Eudragit_RS	25 ug/ml	24h	6	3	3	Homo sapiens	HMEC_50perc_confluence	Human mammary epithelial cells, 50% confluency	In vitro	IV	Hussien et al. 2013^94^	10.1002/phy2.27	Agilent	DNA microarray	https://www.ncbi.nlm.nih.gov/geo/query/acc.cgi?acc=GSE45598	GSE45598_metadata.txt, GSE45598_Unfiltered_DEG.xlsx, Normalized_Expression_Matrix_GSE45598.txt
GSE4567	control, UFP	100 ug/ml	4h	8	4	4	Homo sapiens	HPAEC	Human pulmonary artery endothelial cells	In vitro	IV	Karoly et al. 2007^95^	10.1289/ehp.9556	Affymetrix	DNA microarray	https://www.ncbi.nlm.nih.gov/geo/query/acc.cgi?acc=GSE4567	GSE4567_metadata.txt, Corrected_Expression_Matrix_GSE4567.txt, GSE4567_Filtered_DEG.xlsx, GSE4567_Unfiltered_DEG.xlsx, Normalized_Expression_Matrix_GSE4567.txt
GSE45868	control, Eudragit_RS	25 ug/ml	24h	6	3	3	Homo sapiens	HMEC_90perc_confluence	Human mammary epithelial cells, 90% confluency	In vitro	IV	Hussien et al. 2013^94^	10.1002/phy2.27	Agilent	DNA microarray	https://www.ncbi.nlm.nih.gov/geo/query/acc.cgi?acc=GSE45868	GSE45868_metadata.txt, GSE45868_Filtered_DEG.xlsx, GSE45868_Unfiltered_DEG.xlsx, Normalized_Expression_Matrix_GSE45868.txt
GSE48087	Ag10_Citrate, Ag10_PVP, Ag75_Citrate, Ag75_PVP, control_Citrate, control_HBSS	0.5 ppm	18h	24	4	4	Rattus norvegicus	N24	Rat dopaminergic neurons	In vitro	IV	Chorley et al. 2014^96^	10.1016/j.neuro.2014.08.010	Illumina	DNA microarray	https://www.ncbi.nlm.nih.gov/geo/query/acc.cgi?acc=GSE48087	GSE48087_metadata.txt, Corrected_Expression_Matrix_GSE48087.txt, GSE48087_Filtered_DEG.xlsx, GSE48087_Unfiltered_DEG.xlsx, Normalized_Expression_Matrix_GSE48087.txt
GSE50176	control, rCNT, tCNT	18 mg/m3, 7.2 mg/m3	0h, 24h	18	2	4	Mus musculus	Lung	Whole organ	In vivo	IV	Rydman et al. 2014^97^	10.1186/s12989-014-0048-2	Agilent	DNA microarray	https://www.ncbi.nlm.nih.gov/geo/query/acc.cgi?acc=GSE50176	GSE50176_metadata.txt, Corrected_Expression_Matrix_GSE50176.txt, GSE50176_Filtered_DEG.xlsx, GSE50176_Unfiltered_DEG.xlsx, Normalized_Expression_Matrix_GSE50176.txt
GSE51636	control, Long_asbestos_fibres, Long_carbon_nanotubes, Short_asbestos_fibres, Short_carbon_nanotubes	25 ng, 5 ng	12weeks	20	4	4	Mus musculus	Diaphragm	Whole organ	In vivo	IV	Chernova et al. 2017^98^	10.1016/j.cub.2017.09.007	Agilent	DNA microarray	https://www.ncbi.nlm.nih.gov/geo/query/acc.cgi?acc=GSE51636	GSE51636_metadata.txt, GSE51636_Filtered_DEG.xlsx, GSE51636_Unfiltered_DEG.xlsx, Normalized_Expression_Matrix_GSE51636.txt
GSE51661	control, PLGA	500 ug/ml	24h	6	3	3	Homo sapiens	HUVEC	Human umbilical vein endothelial cells	In vitro	IV	Aday et al. 2014^75^	10.1039/C4RA04041D	Agilent	DNA microarray	https://www.ncbi.nlm.nih.gov/geo/query/acc.cgi?acc=GSE51661	GSE51661_metadata.txt, GSE51661_Filtered_DEG.xlsx, GSE51661_Unfiltered_DEG.xlsx, Normalized_Expression_Matrix_GSE51661.txt
GSE7010	Chapel_hill_coarse, Chapel_hill_fine, Chapel_hill_UFP, control	250 ug/ml	6h	12	3	3	Homo sapiens	PAEC	Primary airway epithelial cells	In vitro	IV	Huang et al. 2011^99^	10.1080/15287394.2010.516238	Affymetrix	DNA microarray	https://www.ncbi.nlm.nih.gov/geo/query/acc.cgi?acc=GSE7010	GSE7010_metadata.txt, Corrected_Expression_Matrix_GSE7010.txt, GSE7010_Filtered_DEG.xlsx, GSE7010_Unfiltered_DEG.xlsx, Normalized_Expression_Matrix_GSE7010.txt
GSE79766	Ag_ions, AgNPs, control	5 ug/ml	24h	9	3	3	Mus musculus	mESC	Mouse embryonic stem cells	In vitro	IV	Gao et al. 2017^100^	10.1186/s12951-017-0265-6	Affymetrix	DNA microarray	https://www.ncbi.nlm.nih.gov/geo/query/acc.cgi?acc=GSE79766	GSE79766_metadata.txt, GSE79766_Filtered_DEG.xlsx, GSE79766_Unfiltered_DEG.xlsx, Normalized_Expression_Matrix_GSE79766.txt
GSE82062	control, nanoSiO2	5 ug/ml	40passage	6	3	3	Homo sapiens	BEAS-2B	Bronchial epithelial cells	In vitro	IV	Guo et al. 2017^101^	10.1080/17435390.2017.1403658	Affymetrix	DNA microarray	https://www.ncbi.nlm.nih.gov/geo/query/acc.cgi?acc=GSE82062	GSE82062_metadata.txt, GSE82062_Filtered_DEG.xlsx, GSE82062_Unfiltered_DEG.xlsx, Normalized_Expression_Matrix_GSE82062.txt
GSE85711	control_aspiration, control_inhalation, rCNT_aspiration, rCNT_inhalation	10 ug, 7.2 mg/m3	24h	15	3	4	Mus musculus	Lung	Whole organ	In vivo	IV	Kinaret et al. 2017^102^	10.1021/acsnano.6b05652	Agilent	DNA microarray	https://www.ncbi.nlm.nih.gov/geo/query/acc.cgi?acc=GSE85711	GSE85711_metadata.txt, Corrected_Expression_Matrix_GSE85711.txt, GSE85711_Filtered_DEG.xlsx, GSE85711_Unfiltered_DEG.xlsx, Normalized_Expression_Matrix_GSE85711.txt
GSE92563	control, TiO2 (E171)	5 mg/kgbw/day	14d, 21d, 2d, 7d	32	4	4	Mus musculus	colon	Whole organ	In vivo	IV	Proquin et al. 2018^103^	10.1016/j.dib.2017.11.067	Agilent	DNA microarray	https://www.ncbi.nlm.nih.gov/geo/query/acc.cgi?acc=GSE92563	GSE92563_metadata.txt, GSE92563_Filtered_DEG.xlsx, GSE92563_Unfiltered_DEG.xlsx, Normalized_Expression_Matrix_GSE92563.txt
GSE92900	control, Fullerene, Graphite, MWCNT (Baytube), MWCNT (SES), rCNT, tCNT	10 ug	24h	24	3	4	Mus musculus	Lung	Whole organ	In vivo	IV	Kinaret et al. 2017^81^	10.1021/acsnano.6b08650	Agilent	DNA microarray	https://www.ncbi.nlm.nih.gov/geo/query/acc.cgi?acc=GSE92900	GSE92900_metadata.txt, Corrected_Expression_Matrix_GSE92900.txt, GSE92900_Filtered_DEG.xlsx, GSE92900_Unfiltered_DEG.xlsx, Normalized_Expression_Matrix_GSE92900.txt
GSE92987	AuNPs, AuNPs-HDM2, control	50 uM	24h	9	3	3	Homo sapiens	Y79	Retinoblastoma	In vitro	IV	Kalmodia et al. 2017^104^	10.1016/j.omtn.2017.10.012	Affymetrix	DNA microarray	https://www.ncbi.nlm.nih.gov/geo/query/acc.cgi?acc=GSE92987	GSE92987_metadata.txt, GSE92987_Filtered_DEG.xlsx, GSE92987_Unfiltered_DEG.xlsx, Normalized_Expression_Matrix_GSE92987.txt
GSE96720	control, Graphene quantum dots (GQD)	50 ug/ml	24h	6	3	3	Homo sapiens	HET1A	Esophagus, epithelial	In vitro	IV	Li et al. 2018^105^	10.1093/toxsci/kfy090	Agilent	DNA microarray	https://www.ncbi.nlm.nih.gov/geo/query/acc.cgi?acc=GSE96720	GSE96720_metadata.txt, GSE96720_Filtered_DEG.xlsx, GSE96720_Unfiltered_DEG.xlsx, Normalized_Expression_Matrix_GSE96720.txt
GSE99929_Jurkat	control, GO, GONH2	50 ug/ml	24h	9	3	3	Homo sapiens	Jurkat	T lymphocyte	In vitro	IV	Orecchioni et al. 2017^106^	10.1038/s41467-017-01015-3	Illumina	DNA microarray	https://www.ncbi.nlm.nih.gov/geo/query/acc.cgi?acc=GSE99929	GSE99929_Jurkat_metadata.txt, GSE99929_Jurkat_Filtered_DEG.xlsx, GSE99929_Jurkat_Unfiltered_DEG.xlsx, Normalized_Expression_Matrix_GSE99929_Jurkat.txt
GSE99929_THP1	control, GO, GONH2	50 ug/ml	24h	9	3	3	Homo sapiens	THP-1	Monocyte	In vitro	IV	Orecchioni et al. 2017^106^	10.1038/s41467-017-01015-3	Illumina	DNA microarray	https://www.ncbi.nlm.nih.gov/geo/query/acc.cgi?acc=GSE99929	GSE99929_THP1_metadata.txt, GSE99929_THP1_Filtered_DEG.xlsx, GSE99929_THP1_Unfiltered_DEG.xlsx, Normalized_Expression_Matrix_GSE99929_THP1.txt
GSE155027	Ag2S, Control, Li-doped Ag2S	160 ug/ml, 320 ug/ml	24h	15	3	3	Homo sapiens	HaCaT	Keratinocytes	In vitro	II	Kang et al. 2020^107^	10.1038/s41598-020-70415-1	Illumina NovaSeq 6000	RNA-Seq	https://www.ncbi.nlm.nih.gov/geo/query/acc.cgi?acc=GSE155027	GSE155027_Filtered_DEG.xlsx, GSE155027_metadata.txt, GSE155027_Unfiltered_DEG.xlsx, Normalized_Count_Matrix_GSE155027.txt
GSE143717	AgNPs_branched_polyethylenimine, AgNPs_Citrate, AgNPs_polyethylene_glycol, Control	1.2 ug/ml	6h, 12h, 24h	32	2	3	Homo sapiens	HepG2	Hepatocyte-like	In vitro	III	House et al. 2020^108^	10.1002/smll.202000299	Illumina HiSeq 2500	RNA-Seq	https://www.ncbi.nlm.nih.gov/geo/query/acc.cgi?acc=GSE143717	GSE143717_Filtered_DEG.xlsx, GSE143717_metadata.txt, GSE143717_Unfiltered_DEG.xlsx, Normalized_Count_Matrix_GSE143717.txt
GSE101992	Control, Silica NPs (MSN_PEG500), Silica NPs (MSN500), Silica NPs (Stober50), Silica NPs (Stober500)	111.8 ug/ml, 13.1 ug/ml, 16.8 ug/ml, 84 ug/ml	4h	15	3	3	Mus musculus	RAW264.7	Macrophage-like	In vitro	IV	Yazdimamaghani et al. 2020^109^	10.1016/j.nano.2017.11.021	Illumina HiSeq 2000	RNA-Seq	https://www.ncbi.nlm.nih.gov/geo/query/acc.cgi?acc=GSE101992	GSE101992_Filtered_DEG.xlsx, GSE101992_metadata.txt, GSE101992_Unfiltered_DEG.xlsx, Normalized_Count_Matrix_GSE101992.txt
GSE125742_HBMSC	Control, Hydroxyapatite	1 ug/ml	24h	7	3	4	Homo sapiens	HBMSC	Human bone marrow derived stem cells	In vitro	IV	Wu et al. 2019^110^	10.1016/j.biomaterials.2019.03.019	HiSeq X Ten	RNA-Seq	https://www.ncbi.nlm.nih.gov/geo/query/acc.cgi?acc=GSE125742	GSE125742_HBMSC_Filtered_DEG.xlsx, GSE125742_HBMSC_metadata.txt, GSE125742_HBMSC_Unfiltered_DEG.xlsx, Normalized_Count_Matrix_GSE125742_HBMSC.txt
GSE125742_HUVEC	Control, Hydroxyapatite	1 ug/ml	24h	6	3	3	Homo sapiens	HUVEC	Human primary umbilical vein endothelial cells	In vitro	IV	Wu et al. 2019^110^	10.1016/j.biomaterials.2019.03.019	HiSeq X Ten	RNA-Seq	https://www.ncbi.nlm.nih.gov/geo/query/acc.cgi?acc=GSE125742	GSE125742_HUVEC_metadata.txt, GSE125742_HUVEC_Unfiltered_DEG.xlsx, Normalized_Count_Matrix_GSE125742_HUVEC.txt
GSE125742_mouse_bone	Control, Hydroxyapatite	20 mg/kgbw	24h	5	2	3	Mus musculus	Bone	Whole organ	In vivo	IV	Wu et al. 2019^110^	10.1016/j.biomaterials.2019.03.019	HiSeq X Ten	RNA-Seq	https://www.ncbi.nlm.nih.gov/geo/query/acc.cgi?acc=GSE125742	GSE125742_mouse_bone_Filtered_DEG.xlsx, GSE125742_mouse_bone_metadata.txt, GSE125742_mouse_bone_Unfiltered_DEG.xlsx, Normalized_Count_Matrix_GSE125742_mouse_bone.txt
GSE125742_mouse_heart	Control, Hydroxyapatite	20 mg/kgbw	24h	6	3	3	Mus musculus	Heart	Whole organ	In vivo	IV	Wu et al. 2019^110^	10.1016/j.biomaterials.2019.03.019	HiSeq X Ten	RNA-Seq	https://www.ncbi.nlm.nih.gov/geo/query/acc.cgi?acc=GSE125742	GSE125742_mouse_heart_metadata.txt, GSE125742_mouse_heart_Unfiltered_DEG.xlsx, Normalized_Count_Matrix_GSE125742_mouse_heart.txt
GSE125742_mouse_kidney	Control, Hydroxyapatite	20 mg/kgbw	24h	6	3	3	Mus musculus	Kidney	Whole organ	In vivo	IV	Wu et al. 2019^110^	10.1016/j.biomaterials.2019.03.019	HiSeq X Ten	RNA-Seq	https://www.ncbi.nlm.nih.gov/geo/query/acc.cgi?acc=GSE125742	GSE125742_mouse_kidney_metadata.txt, GSE125742_mouse_kidney_Unfiltered_DEG.xlsx, Normalized_Count_Matrix_GSE125742_mouse_kidney.txt
GSE125742_mouse_liver	Control, Hydroxyapatite	20 mg/kgbw	24h	6	3	3	Mus musculus	Liver	Whole organ	In vivo	IV	Wu et al. 2019^110^	10.1016/j.biomaterials.2019.03.019	HiSeq X Ten	RNA-Seq	https://www.ncbi.nlm.nih.gov/geo/query/acc.cgi?acc=GSE125742	GSE125742_mouse_liver_metadata.txt, GSE125742_mouse_liver_Unfiltered_DEG.xlsx, Normalized_Count_Matrix_GSE125742_mouse_liver.txt
GSE125742_mouse_muscle	Control, Hydroxyapatite	20 mg/kgbw	24h	5	2	3	Mus musculus	Muscle	Whole organ	In vivo	IV	Wu et al. 2019^110^	10.1016/j.biomaterials.2019.03.019	HiSeq X Ten	RNA-Seq	https://www.ncbi.nlm.nih.gov/geo/query/acc.cgi?acc=GSE125742	GSE125742_mouse_muscle_Filtered_DEG.xlsx, GSE125742_mouse_muscle_metadata.txt, GSE125742_mouse_muscle_Unfiltered_DEG.xlsx, Normalized_Count_Matrix_GSE125742_mouse_muscle.txt
GSE125742_mouse_MuscleStemCell	Control, Hydroxyapatite	1 ug/ml	24h	6	3	3	Mus musculus	Muscle stem cell	Skeletal muscle derived stem cells	In vitro	IV	Wu et al. 2019^110^	10.1016/j.biomaterials.2019.03.019	HiSeq X Ten	RNA-Seq	https://www.ncbi.nlm.nih.gov/geo/query/acc.cgi?acc=GSE125742	GSE125742_mouse_MuscleStemCell_metadata.txt, GSE125742_mouse_MuscleStemCell_Unfiltered_DEG.xlsx, Normalized_Count_Matrix_GSE125742_mouse_MuscleStemCell.txt
GSE125742_mouse_PBMC	Control, Hydroxyapatite	20 mg/kgbw	24h	5	2	3	Mus musculus	PBMC	Periferal bloon mononuclear cells	In vivo	IV	Wu et al. 2019^110^	10.1016/j.biomaterials.2019.03.019	HiSeq X Ten	RNA-Seq	https://www.ncbi.nlm.nih.gov/geo/query/acc.cgi?acc=GSE125742	GSE125742_Mouse_PBMC_metadata.txt, GSE125742_mouse_PBMC_Unfiltered_DEG.xlsx, Normalized_Count_Matrix_GSE125742_mouse_PBMC.txt
GSE125742_mouse_spleen	Control, Hydroxyapatite	20 mg/kgbw	24h	6	3	3	Mus musculus	Spleen	Whole organ	In vivo	IV	Wu et al. 2019^110^	10.1016/j.biomaterials.2019.03.019	HiSeq X Ten	RNA-Seq	https://www.ncbi.nlm.nih.gov/geo/query/acc.cgi?acc=GSE125742	GSE125742_mouse_spleen_Filtered_DEG.xlsx, GSE125742_Mouse_spleen_metadata.txt, GSE125742_mouse_spleen_Unfiltered_DEG.xlsx, Normalized_Count_Matrix_GSE125742_mouse_spleen.txt
GSE153419	(GT)6 SWCNT, AAV, Carboxylated SWCNT, Control, Di-2-ANEQEQ, Di-BASC0, Di-BASC1, Di-BASC2, Fura-2, LPS	NA	2h	24	3	3	Mus musculus	SIM-A9	Microglial cell line	In vitro	IV	Yang & Landry 2020^111^	No publication	Illumina NovaSeq 6000	RNA-Seq	https://www.ncbi.nlm.nih.gov/geo/query/acc.cgi?acc=GSE153419	GSE153419_Filtered_DEG.xlsx, GSE153419_metadata.txt, GSE153419_Unfiltered_DEG.xlsx, Normalized_Count_Matrix_GSE153419.txt
GSE86339_C57Bl6	Control, MWCNT	4 mg/kgbw	28d	6	3	3	Mus musculus	lung (C57Bl/6)	Whole organ	In vivo	IV	Frank et al. 2017^112^	10.1016/j.taap.2017.04.019	Illumina HiSeq 1000	RNA-Seq	https://www.ncbi.nlm.nih.gov/geo/query/acc.cgi?acc=GSE86339	GSE86339_C57Bl6_Filtered_DEG.xlsx, GSE86339_C57Bl6_metadata.txt, GSE86339_C57Bl6_Unfiltered_DEG.xlsx, Normalized_Count_Matrix_GSE86339_C57Bl6.txt
GSE86339_DBA2	Control, MWCNT	4 mg/kgbw	28d	6	3	3	Mus musculus	lung (DBA/2)	Whole organ	In vivo	IV	Frank et al. 2017^112^	10.1016/j.taap.2017.04.019	Illumina HiSeq 1000	RNA-Seq	https://www.ncbi.nlm.nih.gov/geo/query/acc.cgi?acc=GSE86339	GSE86339_DBA2_Filtered_DEG.xlsx, GSE86339_DBA2_metadata.txt, GSE86339_DBA2_Unfiltered_DEG.xlsx, Normalized_Count_Matrix_GSE86339_DBA2.txt

**Online-only Table 2:** Physico-chemical characteristics of the nanomaterials included in the collection.

Data set ID	citation	doi	Material type	Short name	Mapping to treatment	Composition	Stabiliser	Coating	Crystal phase	Purity (%)	Impurities	Supplier / Manufacturer	Address	Supplier code	Batch or Lot No.	Nominal Diameter (nm)	Nominal length (micron)	Nominal Specific Surface area (m^2^/g)	Dispersant	TEM diameter (nm)	TEM width (nm) (median)	TEM length (nm) (median)	No. of walls	BET surafce area (m^2^/g)	DLS Mean Diameter (water) (nm)	PDI	DLS Mean Diameter (Medium) (nm)	PDI	Zeta potential (water) (mV)	Zeta potential (medium) (mV)	Description of dispersion	Endotoxins (EU/mg)	Shape description	Material Specification sheet
GSE112780	Snyder-Talkington et al. 2016^49^	10.1080/15287394.2016.1159635	Multi-walled carbon nanotubes	MWCNT	MWCNT	Carbon	None	None	Not applicable	>99.9%	0.78% (Na, 0.41%; Fe, 0.32%)	Hodogaya Chemical Company	Tokyo, Japan	MWNT-7	#05072001K28	60-170	1-19	23	PBS + serum	not applicable	49 ± 13.4	3860 ± 1900	20-50	not provided	not provided	not provided	not applicable	N/A	not applicable	not applicable	not provided	<0.022	long, entangled	http://www.oecd.org/officialdocuments/publicdisplaydocumentpdf/?cote=env/jm/mono(2016)20&doclanguage=en
GSE112780	Snyder-Talkington et al. 2016^49^	10.1080/15287394.2016.1159635	Crocidolite asbestos	Asbestos	crocidolite	Na_2_(FeIII)_2_(FeII)_3_Si_8_O_22_(OH_2_)	None	None	Not applicable	not given	not given	Kalahari Desert	South Africa	not applicable	As per Cheng et al., 1999.	100-550	11.5	17.1	PBS + serum	not applicable	160 – 800	11500 (range: 2000 – 30000)	not applcable	17.1	not provided	not provided	not applicable	N/A	not applicable	not applicable	not provided	<0.022	long, entangled	
GSE29042	Dymacek & Guo, 2011^50^	10.1109/BIBM.2011.76	Multi-walled carbon nanotubes	MWCNT	MWCNT	Carbon	No details at all of the material provided in the paper. Acknowledges Dale Porter and Vince Castranova (NIOSH) for histopathology data so likely similar mateiral to Porter et al., 2010 which used MWNT-7)			not provided	not provided	not provided	not provided	not provided	not provided	not provided	not provided	not provided	not provided	not provided	not provided	not provided	not provided	long, entangled										
GSE35193	Bourdon et al. 2012^51^	10.1093/toxsci/kfs119	Carbon black	CNP	Carbon black (Printex90)	Carbon	None	None	Not applicable	99	N, H	Evonik Degussa GmbH	Germany	Printex90	not provided	14	not applicable	350	Vehicle (0.9% NaCl MilliQ Water + 10% vol/ Acellular BAL)	not provided	not provided	Not applicable	not applcable	295-338	not provided	not provided	2600	1	not provided	not provided	chains of particles	142	spherical	https://www.thecarycompany.com/carbon-black-printex-90-pwd#specifications
GSE41041	Husain et al. 2013^52^	10.1016/j.taap.2013.03.018	Titanium dioxide	TiO_2_	nanoTiO2	TiO_2_	None	Polyalcohol	Rutile	77.68%	Silicon (5.61%), aluminum (2.42%), zirconium (0.86%) and polyalcohol	Kemira	Pori, Finland	UV-Titan L181	not provided	20.6	not applicable	107.7	0.9% NaCl and 10% acellular BAL fluid	Not determined	Not applicable	not applicable	not provided	not provided	not provided	not provided	not provided	not provided	not provided	not provided	not provided			
GSE42066, GSE42067, GSE42068	Tilton et al. 2014^53^	10.3109/17435390.2013.803624	TiO2 nanobelts	TiO2-NB	TiO2 nanobelt	TiO_2_	None	None	100% anatase	not given	not given	Synthesized as described in Hamilton et al. 2009	not applicable	not applicable	not provided	60-300	15-30	not given	SEGM with 10% FBS	10	Not applicable	Not applicable	not applicable	17.94	2897 ± 117	not provided	not provided	not provided	−30.3 ± 2.8	−11.6 ± 1.0	not provided	not provided	long, entangled	https://particleandfibretoxicology.biomedcentral.com/articles/10.1186/1743-8977-6-35
GSE42066, GSE42067, GSE42068	Tilton et al. 2014^53^	10.3109/17435390.2013.803624	MWCNTs	MWCNTs	MWCNT	Carbon	None	None	Not applicable	95	Residual metal catalysts and carbonaceous impurities removed using a microwave-induced controlled method (Chen and Mitra 2008)	Cheap Tubes Inc.	Brattleboro, VT, USA	not provided	not provided	20-30	10-30	110	DMEM with 10% FBS	Not applicable	20-30	5-10	not given	513	858 ± 58	not provided	not provided	not provided	−11.8 ± 1.1	−10.64 ± 1.0	not provided	not provided	long, entangled	https://www.cheaptubes.com/product/multi-walled-carbon-nanotubes-20-30nm/
GSE51186	Ronzani et al. 2014^54^	10.1093/jac/dku122	Eudragit RL / PVP 3-layer emulsion NPs	Eudragit	Eudragit RL	ethylacrylate and methylmethacrylate	quaternary ammonium groups (0.5-0.8%)	PVP	Not applicable	not given	not given	Evonik Polymers	Darmstadt, Germany	Eudragit® RL PO (MW = 150,000)	Not given	not given	not given	not given	RPMI 1640 +10% FBS	142 ± 60	Not applicable	Not applicable	Not applicable	not provided	not provided	not provided	185 ± 70	not provided	0.190 ± 0.023	+56 ± 3	somewhat polydisperse	not provided	spherical	10.1007/s10565-014-9275-4
GSE55286	Poulsen et al. 2015^55^	10.1016/j.taap.2014.12.011	Multi-walled CNT	CNT_Small_ (NRCWE-026)	MWCNT (NRCWE-26)	Carbon	None	None	Not applicable	87	Al2O3 (14.97%), Fe2O3 (0.29%), CoO (0.11%)	Nanocyl	Sambreville, Belgium	NC7000 CNT	not provided	11 ± 4.5	0.8 ± 0.1	245.8	2% serum collected from C57BL/6 mice	not provided	not provided	not provided	not provided	not provided	not provided	not provided	not provided	not provided	not provided	not provided	not provided	not provided	short	
GSE55286	Poulsen et al. 2015^55^	10.1016/j.taap.2014.12.011	Multi-walled CNT	CNT_Large_	MWCNT (NM-401)	Carbon	None	None	Not applicable	97	P2O5 (0.14%), CO3 (0.08%) and Fe2O3 -0.05%	IO-LE-TECNanomaterials	Supplied by JRC, Ispra	CP-0006-SG	NM-401 (Mitsui-7)	67 ± 26.2	4 ± 0.4	14.6	2% serum collected from C57BL/6 mice	not provided	not provided	not provided	not provided	not provided	not provided	not provided	not provided	not provided	not provided	not provided	not provided	not provided	long, entangled	
GSE55349	Bajak et al. 2015^56^	10.1016/j.toxlet.2014.12.008	Gold nanoparticles	Au-5	Au5	Au	citrate	None	Not applicable	not given	not given	In-house synthesized	JRC	As per Coradeghini et al., 2013; Turkevich et al., 1951).	5	not applicable	not given	DMEM +10% FBS	4.8 ± 1.0	Not applicable	Not applicable	Not applicable	Not applicable	7.5	not given	not provided	not provided	-30	−13.5	Stable dispersion	not provided	spherical		
GSE55349	Bajak et al. 2015^56^	10.1016/j.toxlet.2014.12.008	Gold nanoparticles	Au-30	Au30	Au	citrate	None	Not applicable	not given	not given	In-house synthesized	JRC	As per Coradeghini et al., 2013; Turkevich et al., 1951).	30	not applicable	not given	DMEM +10% FBS	32.2 ± 1.0	Not applicable	Not applicable	Not applicable	Not applicable	37.3	not given	not provided	not provided	not provided	−13.5	Stable dispersion	not provided	spherical		
GSE60799	Halappanavar et al. 2014^57^	10.1002/em.21936	Titanium dioxide	TiO_2_NP^10.5^	nanoTiO2 (NRCWE‐030)	TiO2	None	None	Rutile	98	<1%	NanoAmor	provided via NRCWE	not applicable	NRCWE‐030	10.5	not applicable	139.1	2% serum in water	not provided	not provided	not provided	not applicable	not provided	126 ± 2	0.157	not provided	not provided	not provided	not provided	Stable dispersion	not provided	slighly elongated	Can’t find this size particels for rutile in current listing
GSE60799	Halappanavar et al. 2014^57^	10.1002/em.21936	Titanium dioxide	TiO_2_NP^38^	nanoTiO2 (NRCWE‐025)	TiO2	None	None	Rutile	98	<1%	Nabond	provided via NRCWE	not applicable	NRCWE‐025	38	not applicable	28.2	2% serum in water	not provided	not provided	not provided	not applicable	not provided	214 ± 6	0.172	not provided	not provided	not provided	not provided	Stable dispersion	not provided	spherical	Orignal specifiction sheet not located online
GSE60798	Halappanavar et al. 2014^57^	10.1002/em.21936	Titanium dioxide	TiO_2_NP^10^	nanoTiO2 (NRCWE‐001)	TiO2	None	None	Rutile	98	<1%	NanoAmor	provided via NRCWE	not applicable	NRCWE‐001 (no charge)	10	not applicable	99	2% serum in water	not provided	not provided	not provided	not applicable	not provided	108 ± 1	0.145	not provided	not provided	not provided	not provided	Stable dispersion	not provided	spherical	Can’t find this size particels for rutile in current listing
GSE60798	Halappanavar et al. 2014^57^	10.1002/em.21936	Titanium dioxide	TiO_2_NP^10+^	nanoTiO2 (NRCWE‐002)	TiO2	None	None	Rutile	98	<1%	NanoAmor	provided via NRCWE	not applicable	NRCWE‐002 (Positively charged)	10	not applicable	84	2% serum in water	not provided	not provided	not provided	not applicable	not provided	1719 ± 166	0.362	not provided	not provided	not provided	not provided	Highly agglomerated /Unstable dispersion.	not provided	roughly spherical	
GSE63552	Nymark et al. 2015^58^	10.3109/17435390.2015.1017022	MWCNT	MWCNT	MWCNT (Mitsui7)	Carbon	None	None	N/A	>99.9%	Fe, Si, Na, Mg, Ca, O	Mitsui & Co., Ltd.,	Tokyo, Japan	MWCNT-7	not stated	60-170	1-19	23	BEGM with 0.6 mg/ml BSA	not applicable	180	4600	not provided	8.3	not provided	not provided	not provided	not provided	not provided	not provided	rapid sedimentation	not provided	long, entangled	
GSE63552	Nymark et al. 2015^58^	10.3109/17435390.2015.1017022	standard reference crocidolite asbestos	crocidolite	crocidolite	Na_2_(FeIII)_2_(FeII)_3_Si_8_O_22_(OH_2_)	None	None	N/A	not given	Si, Al, O, Na, Mg, K, Ca	UICC (Union for International Cancer Control	Geneva Switzerland	not stated	Lot# 05072001K28	not applicable	not applicable	not applicable	BEGM with 0.6 mg/ml BSA	not applicable	1100 ± 500	21000 ±1800	not applicable	1.07	not provided	not provided	not provided	not provided	not provided	not provided	rapid sedimentation	not provided	long, entangled	
GSE63552	Nymark et al. 2015^58^	10.3109/17435390.2015.1017022	non-carcinogenic glass wool (Neg Contol)	glass wool	glass wool	SiO2	None	None	N/A	not given	C	Dr David Brown, Heriot Watt University	MMVF10	not stated		not applicable		BEGM with 0.6 mg/ml BSA	not applicable	74 ± 28	5700 ± 3700	not applicable	22	not provided	not provided	not provided	not provided	not provided	not provided	rapid sedimentation	not provided	long, entangled		
GSE63806	Pisani et al. 2015^59^	10.1186/s12864-015-1521-5	Silica	AEROSIL® 200	nanoSiO2	SiO_2_	None	None	hydrophilic fumed silica	99.8	SiO2	Evonik	Germany	not stated	not stated	12	not applicable	200	DMEM/F12 with 10% FBS	not provided	not provided	not provided	not provided	not provided	10 ± 4	not provided	160 ± 90	not provided	not provided	not provided	not provided	not provided	disks with diameter 10 ± 5 nm and thickness 2 ± 0.3	
GSE81569	Rahman et al. 2017^60^	10.1093/mutage/gew048	Anatase TiO2	aTiO_2_^8^	nanoTiO2 (anatase)	TiO2	None	None	Anatase	not given	Al, Si, Na, P, S, Zr	Sachtleben Chemie GmbH	Germany	Hombikat UV100	Actually got from OECD programme	8	not applicable	> 250	MilliQ water	8	Not applicable	Not applicable	not applicable	234.47–229.00	148.4 ± 38.3	not provided	not provided	not provided	not provided	not provided	not provided	not provided	spherical	http://kuroppe.tagen.tohoku.ac.jp/~dsc/0075e072.pdf
GSE81564	Rahman et al. 2017^60^	10.1093/mutage/gew048	Anatase TiO2	aTiO_2_^20^	nanoTiO2 (anatase)	TiO2	None	None	Anatase	99%	none	Cristal Global (http://www.cristal.com)	PC105	Actually got from OECD programme	30	not applicable		MilliQ water	~30	~35–70	not provided	not applicable	90	242.9 ± 53.1	not provided	not provided	not provided	not provided	not provided	not provided	not provided	elongated		
GSE81568	Rahman et al. 2017^60^	10.1093/mutage/gew048	Anatase TiO2	aTiO_2_^300^	nanoTiO2 (anatase)	TiO2	None	None	Anatase	99%	Al, Zr, polyalcohol	Cristal Global (http://www.cristal.com)	tiona	Actually got from OECD programme	300	not applicable		MilliQ water	100–120	Not applicable	Not applicable	not applicable	10	130.1 ± 49.8	not provided	not provided	not provided	not provided	not provided	not provided	not provided	spherical		
GSE81567	Rahman et al. 2017^60^	10.1093/mutage/gew048	Mixed anatase and rutile TiO2	raTiO_2_	nanoTiO2 (rutile/anatase mix)	TiO2	None	None	81.5:18.5 Rutile/anatase	99%	none	Evonik Degussa GmbH	Germany	P25	Actually got from OECD programme	22/40	not applicable		MilliQ water	18-52	Not applicable	Not applicable	not applicable	52.81–55.49	242.9 ± 56.4	not provided	not provided	not provided	not provided	not provided	not provided	not provided	spherical	
GSE81565	Rahman et al. 2017^60^	10.1093/mutage/gew048	Rutile Titanium dioxide with hydrophillic coating	rTiO_2_ (HY)	nanoTiO2 (rutile hydrophilic)	TiO2	None	Modified Dimethicone (2 wt%)	Rutile	91.3	Al, Si, Fe and S	Sachtleben Chemie GmbH	Germany	UV Titan M262	Actually got from OECD programme	20	not applicable		MilliQ water	18-52	Not applicable	Not applicable	not applicable	51.69–50.8	110.1 ± 27.9	not provided	not provided	not provided	not provided	not provided	not provided	not provided	spherical	
GSE81566	Rahman et al. 2017^60^	10.1093/mutage/gew048	Rutile Titanium dioxide with hydrophobic coating	rTiO_2_ (HP)	nanoTiO2 (rutile hydrophobic)	TiO2	None	Modified Glycerol (1-2 wt %)	Rutile	92.7	Al, Si, S	Sachtleben Chemie GmbH	Germany	UV Titan M212	Actually got from OECD programme	21	not applicable		MilliQ water	~30	~35–70	not provided	not applicable	57.07–57.18	39.3 ± 7.3	not provided	not provided	not provided	not provided	not provided	not provided	not provided	elongated	
GSE98236	Pisani et al. 2017^61^	10.1080/17435390.2017.1378749	Magnetic mesoporous silica	M-MSNs	M-MP-Silica NP (Pristine)	Fe_3_O_4_ core and SiO_2_ shell	None	None	amorphous	not provided	not provided	In-house synthesized as per Nyalosaso et al. (2016)	Not applicable	Not applicable	not provided	not applicable	not given	HBS (20mM Hepes, 5mM NaCl) or 1X PBS at pH 7.4 and 20 °C.	117 ±2	not applicable	not applicable	not applicable	not provided	172 ±6	0.17	not provided	not provided	-39.1 ±1.4	not provided	not provided	not provided	spherical		
GSE98236	Pisani et al. 2017^61^	10.1080/17435390.2017.1378749	Magnetic mesoporous silica	M-MSNs	M-MP-Silica NP (PEG)	Fe_3_O_4_ core and SiO_2_ shell	None	PEG	amorphous	not provided	not provided	In-house synthesized as per Nyalosaso et al. (2016)	Not applicable	Not applicable	not provided	not applicable	not given	HBS (20mM Hepes, 5mM NaCl) or 1X PBS at pH 7.4 and 20 °C.	123 ±3	not applicable	not applicable	not applicable	not provided	156 ±1	0.18	not provided	not provided	-30.4 ±2.9	not provided	not provided	not provided	spherical		
GSE98236	Pisani et al. 2017^61^	10.1080/17435390.2017.1378749	Magnetic mesoporous silica	M-MSNs	M-MP-Silica NP (DMPC)	Fe_3_O_4_ core and SiO_2_ shell	None	DMPC	amorphous	not provided	not provided	In-house synthesized as per Nyalosaso et al. (2016)	Not applicable	Not applicable	not provided	not applicable	not given	HBS (20mM Hepes, 5mM NaCl) or 1X PBS at pH 7.4 and 20 °C.	132 ± 4	not applicable	not applicable	not applicable	not provided	180 ± 2	0.12	not provided	not provided	-10.3 ± 0.4	not provided	not provided	not provided	spherical		
GSE122197	Ilves et al. 2019^62^	10.1186/s12989-019-0309-1	Copper oxide	CuO	CuO	CuO	none	none	monoclinic	99	not provided	PlasmaChem GmbH	Berlin, Germany	NanoSolutions	YF1309121	10-20	not applicable	42 ± 2	Krebs	12.00 ± 0.37	not applicable	not applicable	not applicable	not provided	41 ± 28	not provided	62 ± 50	not provided	14.0 ± 1.2	not provided	not provided	not provided	spherical	
GSE122197	Ilves et al. 2019^62^	10.1186/s12989-019-0309-1	Copper oxide	CuO COOH	CuO_COOH	CuO	COOH	none	monoclinic	99	not provided	PlasmaChem GmbH	Berlin, Germany	NanoSolutions	YF140114	10-20	not applicable	7.4 ± 0.5	Krebs	6.45 ± 0.16	not applicable	not applicable	not applicable	not provided	121 ± 91	not provided	128 ± 58	not provided	- 7.3 ± 0.5	not provided	not provided	not provided	spherical	
GSE122197	Ilves et al. 2019^62^	10.1186/s12989-019-0309-1	Copper oxide	CuO NH3	CuO_NH3	CuO	NH3	none	monoclinic	99	not provided	PlasmaChem GmbH	Berlin, Germany	NanoSolutions	YF140114	10-20	not applicable	6.1 ± 0.5	Krebs	9.53 ± 0.22	not applicable	not applicable	not applicable	not provided	46 ± 36	not provided	96 ± 75	not provided	27.7 ± 0.5	not provided	not provided	not provided	spherical	
GSE122197	Ilves et al. 2019^62^	10.1186/s12989-019-0309-1	Copper oxide	CuO PEG	CuO_PEG	CuO	PEG	none	monoclinic	99	not provided	PlasmaChem GmbH	Berlin, Germany	NanoSolutions	YF140114	10-20	not applicable	not given	Krebs	7.46 ± 0.42	not applicable	not applicable	not applicable	not provided	100 ± 36	not provided	189 ± 113	not provided	- 16.8 ± 0.4	not provided	not provided	not provided	spherical	
GSE122197	Kooter et al. 2019^63^	10.1021/acsnano.9b01823	Copper oxide	nCuO	CuO	CuO	none	none	monoclinic	99	not provided	PlasmaChem GmbH	Berlin, Germany	NanoSolutions	not stated	10-20	not applicable	10-2po1ee	Air with 50% relative humidity	not provided	not applicable	not applicable	not applicable	55	not provided	not provided	not provided	not provided	not provided	not provided	not provided	not provided	spherical	
GSE122197	Kooter et al. 2019^63^	10.1021/acsnano.9b01823	Copper oxide	nCuO^COOH^	CuO_COOH	CuO	COOH	none	monoclinic	99	not provided	PlasmaChem GmbH	Berlin, Germany	NanoSolutions	not stated	10-20	not applicable	10-2po1ee	Air with 50% relative humidity	not provided	not applicable	not applicable	not applicable	55	not provided	not provided	not provided	not provided	not provided	not provided	not provided	not provided	spherical	
GSE30200	Sellamuthu et al. 2011^65^	10.3109/08958378.2011.625995	Min-U-Sil-5 crystalline silica	Silica	Silica	SiO2	None	None	Crystalline	not stated	not stated	US Silica	Berkeley Springs, WV	Min-U-Sil-5 c	not stated	not stated	not stated	not stated	Serum-free F-12K	not stated	not stated	not stated	not applicable	not provided	not provided	not provided	not provided	not provided	not provided	not provided	not provided	Endotoxin-free	spherical	
GSE46998	Søs Poulsen et al. 2013^66^	10.1371/journal.pone.0080452	Multi-walled carbon nanotubes (MWCNTs)	MWCNTs	MWCNT	Carbon	None	None	N/A	99%	not stated	Hadoga Chemical industry (formerly Mitsui)	MWCNT- XNRI-7 (also called Mitsui7)	05072001K28	100	not applicable	not stated	0.9% NaCl and 10% acellular BAL fluid	74.4 ± 27.2	100	3860 (27.5% of particles > 5000)	not provided	not provided	5000	1	not provided	not provided	not provided	not provided	good	not provided	Tubes: intertwined and agglomerated, with both long and straight, and bendy	slightly skewed unimodal	
GSE62253	Böhmert et al. 2015^67^	10.3109/17435390.2014.980760	Silver NPs	AgPURE	NanoAg	Ag	4% Tagat T and 4% Tween 20	None	N/A	not stated	not stated	Rent a Scientist GmbH	Regensburg, Germany	similar to BAM001 and NM-300	not stated	not provided	not applicable	not stated	DMEM + 10% heat inactivated FBS	15	N/A	N/A	not applicable	not provided	28	not provided	not provided	not provided	not provided	not provided	5% Ag release in 24 hours for 5 mg/mL in cell culture medium	not provided	spherical	
GSE62769	Perkins et al. 2015^68^	10.1093/hmg/ddu551	crocidolite asbestos fibers	crocidolite asbestos	asbestos		none	None	Crocidolite	not stated	not stated	NIEHS reference sample	South Africa	not stated	not stated	not provided	not provided	not stated	Hank’s buffered saline solution (HBSS)	not provided	not provided	not provided	not applicable	not provided	not provided	not provided	not provided	not provided	not provided	not provided	not provided	not provided	Tubes	
GSE62769	Perkins et al. 2015^68^	10.1093/hmg/ddu551	cristobalite silica particle	cristobalite silica	Silica	SiO2	none	None	Cristobalite	95.5 ± 0.03	not stated	C&E Mineral Corp	King of Prussia, PA, USA	not stated	not stated	2016 ± 200	not applicable	5.1	Hank’s buffered saline solution (HBSS)	not provided	not provided	not provided	not applicable	not provided	not provided	not provided	not provided	not provided	not provided	not provided	not provided	not provided	Tubes	
GSE75429	Rahman et al. 2017^69^	10.1016/j.mrgentox.2017.08.005	Multi-walled CNT	Mitsui-7	MWCNT (Mitsui7)	Carbon	none	None	N/A	not stated	<1 (Na, Mn, Al, Ni, Mg (Na: 499 ± 103 μg/g, Mg: 1 ± 1 μg/g, Al: 66 ± 19 μg/g, Fe: 355 ± 2 μg/g, Ni: 1 μg/g)	Mitsui’ & Company	Japan	not stated	not stated	40-90	not applicable	24-28	0.05% BSA in water	N/A	74 ± 28	5700 ± 3700	not provided	28	not provided	not provided	682 ± 13	not provided	not provided	not provided	not provided	not provided	Tubes	
GSE75429	Rahman et al. 2017^69^	10.1016/j.mrgentox.2017.08.005	Multi-walled CNT	NM-401	MWCNT (NM-401)	Carbon	none	None	N/A	not stated	<5 Na, Mn, Al, Ni, Mg (Na: 581 ± 32 μg/g, Fe: 379 ± 71 μg/g, Mg: 0 ± 0.3 μg/g, Al: 59 ± 4 μg/g, Ni: 2 μg/g)	IO-LE-TEC Nanomaterials (CP-0006-SG)	not stated	not stated	10-30	5-15	40-300	0.05% BSA in water	N/A	67 ± 26.2	4000 ± 2400	not provided	18	not provided	not provided	710 ± 17	not provided	not provided	not provided	not provided	not provided	Tubes		
GSE16727	Busch et al. 2010^70^	10.1186/1471-2164-11-65	Tungsten carbide	WC	WC	WC	none	None	N/A	not provided	not provided	In-house produced from powder by chemical process and deaggregated and mixed via ball mill	not stated	not applicable	not applicable	not applicable	RPMI + 5% FBS	not given	not provided	not provided	not applicable	6.9	not provided	not provided	145 ± 5	0.2	-35	-20	stable in culture medium for 1 week	not provided	spherical			
GSE16727	Busch et al. 2010^70^	10.1186/1471-2164-11-65	Tungsten carbide cobalt	WC-Co	WC-Co	WC-Co	none	None	N/A	not provided	not provided	In-house produced from powder by chemical process and deaggregated and mixed via ball mill	not stated	not applicable	not applicable	not applicable	RPMI + 5% FBS	not given	not provided	not provided	not applicable	6.6	not provided	not provided	145 ± 5	0.2	-50	-23	stable in culture medium for 1 week	not provided	spherical			
GSE39330_jurkat	Tuomela et al. 2013^71^	10.1371/journal.pone.0068415	Zinc oxide	ZnO-1	ZnO	ZnO	none	unknown	N/A	not provided	not provided	IBU-tec advanced materials AG	Weimar, Germany	not stated	12	not applicable	60 ± 5	Sonication in ultra-pure water, prepared immediately before use	14.7 ± 1.07	N/A	N/A	not applicable	not provided	159±3	0.15	153±4	0.12	-23.9±0.2	-17.3±0.1	not provided	not provided	spherical		
GSE39330_jurkat	Tuomela et al. 2013^71^	10.1371/journal.pone.0068415	Zinc oxide	ZnO-2	mandelic_acid_ZnO	ZnO	none	mandelic acid	N/A	not provided	not provided	In-house functionalisation of ZnO-1	Not applicable	Not applicable	Not applicable	12	not applicable	60 ± 5	Sonication in ultra-pure water, prepared immediately before use	11.7±0.77	N/A	N/A	not applicable	not provided	312±3	0.1	287±2	0.12	-9.9±0.2	-12.3±0.2	not provided	not provided	spherical	
GSE39330_jurkat	Tuomela et al. 2013^71^	10.1371/journal.pone.0068415	Zinc oxide	ZnO-3	mercaptopropyl_trimethoxysilane_ZnO	ZnO	none	mercaptopropyl-trimethoxysilane	N/A	not provided	not provided	In-house functionalisation of ZnO-1	Not applicable	Not applicable	Not applicable	12	not applicable	60 ± 5	Sonication in ultra-pure water, prepared immediately before use	14.6±1.2 (core) 10.9±0.65 (shell)	N/A	N/A	not applicable	not provided	548±4	0.14	341±5	0.14	-23.3±0.4	-23.3±0.4	not provided	not provided	spherical	
GSE39330_jurkat	Tuomela et al. 2013^71^	10.1371/journal.pone.0068415	Zinc oxide	ZnO-4	methoxyl_ZnO	ZnO	none	methoxyl	N/A	not provided	not provided	In-house functionalisation of ZnO-1	Not applicable	Not applicable	Not applicable	12	not applicable	60 ± 5	Sonication in ultra-pure water, prepared immediately before use	7.8±0.88	N/A	N/A	not applicable	not provided	387±1	0.14	110±4	0.15	-17.9±0.2	-12.7±0.4	not provided	not provided	spherical	
GSE39330_jurkat	Tuomela et al. 2013^71^	10.1371/journal.pone.0068415	Zinc oxide	ZnO-5	diethylene_glycol_ZnO	ZnO	none	diethylene glycol	N/A	not provided	not provided	In-house functionalisation of ZnO-1	Not applicable	Not applicable	Not applicable	12	not applicable	60 ± 5	Sonication in ultra-pure water, prepared immediately before use	5.4±0.5	N/A	N/A	not applicable	not provided	125±4	0.12	278±3	0.11	19.2±0.2	13.6±0.3	not provided	not provided	spherical	
GSE39330_jurkat	Tuomela et al. 2013^71^	10.1371/journal.pone.0068415	Zinc oxide	ZnO-9	folic_acid_ZnO	ZnO	none	folic acid	N/A	not provided	not provided	In-house functionalisation of ZnO-1	Not applicable	Not applicable	Not applicable	12	not applicable	60 ± 5	Sonication in ultra-pure water, prepared immediately before use	N/A	40.4±2.6	404±8	not applicable	not provided	412±4	0.15	466±3	0.15	-31.7±0.3	-15.4±0.5	not provided	not provided	spherical	
GSE45322	Osmond-McLeod et al. 2013^73^	10.1186/1743-8977-10-54	Zinc oxide uncoated	Zinc oxide uncoated	ZnO_ZCOTE	ZnO	none	uncoated	N/A	not provided	not provided	BASF	Germany	Z-COTE	EHDA3001	70-200	not applicable	12-14	Complete Hepatocyte Medium (HM, HM-5201)	N/A	44 ± 2	73 ± 4	not applicable	13 ± 2	400	0.3	260	0.3	27	8	not provided	Below detection limit	heterogeneous mix of rectangular-shaped particle	
GSE45322	Osmond-McLeod et al. 2013^73^	10.1186/1743-8977-10-54	Zinc oxide uncoated	Zinc oxide uncoated	ZnO_Nanosun	ZnO	none	uncoated	N/A	not provided	not provided	Micronisers	Victoria, Australia	Nanosun P99/30	4051	30-50	not applicable	not available	Complete Hepatocyte Medium (HM, HM-5201)	25 ± 1	N/A	N/A	not applicable	30 ± 3	600	0.6	25	0.7	9	7	not provided	Below detection limit	rod-shaped	
GSE45322	Osmond-McLeod et al. 2013^73^	10.1186/1743-8977-10-54	Zinc oxide	Zinc oxide coated	ZnO_HP1	ZnO	none	triethoxycaprylylsilane	N/A	not provided	not provided	BASF	Germany	Z-COTE HP1	CNHE0602	90-190	not applicable	12-14	Complete Hepatocyte Medium (HM, HM-5201)	N/A	28 ± 2	96 ± 6	not applicable	14.9 ± 0.5	240	0.3	300	0.6	-10	-10	not provided	Below detection limit	heterogeneous mix of rectangular-shaped particle	
GSE45322	Osmond-McLeod et al. 2013^73^	10.1186/1743-8977-10-54	Zinc oxide	Zinc oxide coated	ZnO_MAX	ZnO	none	dimethoxydiphenylsilane/triethoxycaprylylsilane crosspolymer	N/A	not provided	not provided	BASF	Germany	Z-COTE MAX	FCHE1301	36-95	not applicable	not available	Complete Hepatocyte Medium (HM, HM-5201)	N/A	36 ± 2	95 ± 5	not applicable	12 ± 1	broad	1	90	0.2	-7	-6	not provided	Below detection limit	heterogeneous mix of rectangular-shaped particle	
GSE51417	Teeguarden et al. 2014^74^	10.1186/s12989-014-0046-4	Superparamagnetic iron oxide particles (SPIO)	Fe_3_O_4_	SPIO	Iron Oxide	none	N(trimethoxysilylpropyl)ethylenediamine-triacetic	magnetite	not provided	not provided	In-house synthesized	Not applicable	Not applicable	Not applicable	12.8	not applicable	8.87 × 10^−7^ cm^2^ in 1 picogram	DI water	not given	not given	not given	not applicable	not provided			207			-41	good for coated ones	Not given	spherical	
GSE51421	Aday et al. 2014^75^	10.1039/C4RA04041D	Poly(lactic acid-co-glycolic acid)	NP210-PFCE	PLGA	PLGA	None	protamine sulfate (cationic)	N/A	not provided	not provided	In-house synthesized	Not applicable	Not applicable	Not applicable	Not applicable	not applicable		M200 or QBSF-60	not given	not given	not given	not applicable	not provided	210	0.12 + 0.01	not provided	not provided	7.0 ± 1.7	not provided	not provided	Not given	spherical	
GSE53700	Fede et al. 2014^76^	10.3390/ijerph110908867	Silica dioxide	AS30	SiO2_AS30	SiO2	ammounium counterions	None	N/A	not provided	not provided	Sigma Aldrich	St. Louis, MO, USA.	Ludox Silica - AS30	Not given	20	not applicable	~230	PBS	18 ± 3	N/A	N/A	not applicable	not provided	20 ± 4		150 with 3% serum (same as PBS in medium with no serum)	−25.9	not provided	not provided	Not given	spherical		
GSE53700	Fede et al. 2014^76^	10.3390/ijerph110908867	Silica dioxide	SM30	SiO2_SM30	SiO2	sodium counterions	None	N/A	not provided	not provided	Sigma Aldrich	St. Louis, MO, USA.	Ludox Silica - SM30	Not given	10	not applicable	320-400	PBS	9 ± 3	N/A	N/A	not applicable	not provided	14 ± 4		150 with 3% serum (same as PBS in medium with no serum)	−26.3	not provided	not provided	Not given	spherical		
GSE56324, GSE56325	Husain et al. 2015^77^	10.3109/17435390.2014.996192	Titanium dioxide	nano-TiO_2_	nanoTiO2	TiO2	none	Polyalcohol	Rutile	77.68	Si 5.61 wt%; Al 2.42 wt%; Zr 0.86 wt%; Na 0.45 wt %	Kemira	Pori, Finland	UV-Titan L181	Not given	20.6±0.3	not applicable	107.7	10% v/v acellular BAL collected from C57BL/6 mice			not applicable	not provided	4147±1260	0.882	not provided	not provided	not provided	not provided	1362±221 (0.253; 3.1 mm filtered) 1551±406 (0.722; 0.2 mm filtered)	Not given	spherical		
GSE61366	Poulsen et al. 2015^78^	10.1016/j.taap.2015.01.011	Multi-walled CNT	CNT_Small_ (NRCWE-026)	MWCNT (NCRWE-026)	Carbon	none	none	N/A	87	Al2O3 (14.97%), Fe2O3 (0.29%), CoO (0.11%)	Nanocyl	Sambreville, Belgium	NC7000 CNT	Not given	11 ± 4.5	0.8 ± 0.1	245.8	2% serum collected from C57BL/6 mice	not given	not given	not given	not applicable	not provided	not provided	not provided	not provided	not provided	not provided	not provided	not provided	Not given	long tube	
GSE61366	Poulsen et al. 2015^78^	10.1016/j.taap.2015.01.011	Multi-walled CNT	CNT_Large_	MWCNT (NM-401)	Carbon	none	none	N/A	97	P2O5 (0.14%), CO3 (0.08%) and Fe2O3 -0.05%	IO-LE-TECNanomaterials	Supplied by JRC, Ispra	CP-0006-SG	NM-401 (Mitsui-7)	67 ± 26.2	4 ± 0.4	14.6	2% serum collected from C57BL/6 mice	not given	not given	not given	not applicable	not provided	not provided	not provided	not provided	not provided	not provided	not provided	not provided	Not given	long tube	
GSE68036	Husain et al. 2015^79^	10.1016/j.taap.2015.11.003	Carbon black	CBNPs	Carbon black (Printex90)	Carbon	none	none	N/A	99%	< 1% polycyclic aromatic hydrocarbon	Evonik/Degussa	Frankfurt, Germany	Printex 90	Not given	14	not applicable	295–338	0.2 μm filtered, ɤ-irradiated Nanopure Diamond UV water	not given	not given	not given	not applicable	not provided	50-100	not provided	not provided	not provided	not provided	not provided	not provided	142	spherical	
GSE84982_Caco2, GSE84982_MCF7	van der Zande et al. 2016^80^	10.1080/17435390.2016.1225132	Silver NPs	BioPure Ag-20	20nm_AgNPs	Ag	citrate	uncapped	N/A	99.99	not stated	Nanocomposix	San Diego, CA	EAW1091																				
Not given																																		
	20.3±1.9	not applicable	27.6	DMEM + FBS	not given	not given	not given	not applicable	not provided	not provided	not provided	40±4	not provided	not provided	not provided	not provided	<5	spherical																
GSE84982_Caco2, GSE84982_MCF7	van der Zande et al. 2016^80^	10.1080/17435390.2016.1225132	Silver NPs	BioPure Ag-30	30nm_AgNPs	Ag	citrate	uncapped	N/A	99.99	not stated	Nanocomposix	San Diego, CA	CTH1088	Not given	34.4±3.4	not applicable	14.2	DMEM + FBS	not given	not given	not given	not applicable	not provided	not provided	not provided	43±1	not provided	not provided	not provided	not provided	<2.5	spherical	
GSE84982_Caco2, GSE84982_MCF7	van der Zande et al. 2016^80^	10.1080/17435390.2016.1225132	Silver NPs	BioPure Ag-60	60nm_AgNPs	Ag	citrate	uncapped	N/A	99.99	not stated	Nanocomposix	San Diego, CA	EAW1093	Not given																			
61.2±5.3																																		
	not applicable	9.4	DMEM + FBS	not given	not given	not given	not applicable	not provided	not provided	not provided	88±2	not provided	not provided	not provided	not provided	<2.5	spherical																	
GSE84982_Caco2, GSE84982_MCF7	van der Zande et al. 2016^80^	10.1080/17435390.2016.1225132	Silver NPs	BioPure Ag-100	110nm_AgNPs	Ag	citrate	uncapped	N/A	99.99	not stated	Nanocomposix	San Diego, CA	CTH1409	Not given	112.6±7.8	not applicable	5.7	DMEM + FBS	not given	not given	not given	not applicable	not provided	not provided	not provided	135±6	not provided	not provided	not provided	not provided	<2.5	spherical	
GSE92899	Kinaret et al. 2017^81^	10.1021/acsnano.6b08650	long rigid MWCNT	rCNT	rCNT	Carbon	None	None	N/A	99.79 wt%	not stated	Mitsui & Co.	Tokyo, Japan	MWCNT-7	Not given			26	RPMI + 10% FBS	N/A	50	13000	not applicable	22	not given	not given	not given	not given	not given	not given	not given	Not given	tube	
GSE92899	Kinaret et al. 2017^81^	10.1021/acsnano.6b08650	long tangled MWCNT	tCNT	tCNT	Carbon	None	None	N/A	>95	not stated	Cheaptubes Inc.	Grafton, VT, USA	Cheaptubes	Not given	8-15	10-50	233	RPMI + 10% FBS	N/A	11.5	30000	not stated	180	not given	not given	not given	not given	not given	not given	not given	not stated	tube	https://www.cheaptubes.com/product/multi-walled-carbon-nanotubes-8-15nm/
GSE92899	Kinaret et al. 2017^81^	10.1021/acsnano.6b08650	short tangled MWCNT	Baytube	MWCNT (Baytube)	Carbon	None	None	N/A	99	not stated	Bayer Material Science	Laufenburg, Germany	Baytubes C150 HP	Not given	5-20	1-10	not given	RPMI + 10% FBS	N/A	14.5	1000	not stated	204	not given	not given	not given	not given	not given	not given	not given	not stated	tube	Not produced anymore - no specification available
GSE92899	Kinaret et al. 2017^81^	10.1021/acsnano.6b08650	long rigid carbon fiber graphite	graphite	Graphite	Carbon	None	None	N/A	not stated	not stated	Sigma-Aldrich	Finland	636398	Not given	not given	not given	not given	RPMI + 10% FBS	N/A	140	10000	not applicable	32	not given	not given	not given	not given	not given	not given	not given	Not given	fibre	
GSE92899	Kinaret et al. 2017^81^	10.1021/acsnano.6b08650	hollow carbon sphere	fullerene	Fullerene	Carbon	None	None	N/A	not stated	not stated	MTR Ltd.	Cleaveland, OH, USA	MTS60	Not given	1	not applicable	not stated	RPMI + 10% FBS	N/A	100	100	not applicable	20	not given	not given	not given	not given	not given	not given	not given	Not given	sphere	
GSE92899	Kinaret et al. 2017^81^	10.1021/acsnano.6b08650	short rigid MWCNT	SES	MWCNT (SES)	Carbon	None	None	N/A	>95%	not stated	SES Research	Houston, Texas	900−1260	Not given	10-30	1-2		RPMI + 10% FBS	N/A	20	1500	not applicable	60	not given	not given	not given	not given	not given	not given	not given	Not given	tube	https://www.sesres.com/carbon-nanotubes/
GSE88786	Lastra et al. 2019^82^	10.33218/prnano2(4).190802.1	Fe3O4@TiO2	Fe3O4@TiO	Fe3O4_TiO2_NP	Fe3O4@TiO2	None	Dopamine	N/A	not stated	not stated	In-house produced	Not applicable	not provided	Not given	Not given	not applicable	Not given	Not given	Not given	Not given	Not given	not applicable	Not given	90	Not given	Not given	Not given	-27	Not given	stable for > 6 months	Not given	Not given	
EMTAB6396_A549, EMTAB6396_Beas2B, EMTAB6396_MRC9, EMTAB6396_THP1	Scala & Kinaret et al. 2018^83^	10.1016/j.impact.2018.05.003	Carbon black	CBL	Carbon black	Carbon	None	None	N/A	not stated	not stated	Evonik	Germany	Printex 90	not stated	14	14	265	Cell culture medium + 1% FCS	Not given	Not given	Not given	not applicable	not given	not given	not given	not given	not given	not given	not given	not given	Not given	Particle	
EMTAB6396_A549, EMTAB6396_Beas2B, EMTAB6396_MRC9, EMTAB6396_THP1	Scala & Kinaret et al. 2018^83^	10.1016/j.impact.2018.05.003	Fullerene C60	FUL	Fullerene	Carbon	None	None	N/A	not stated	not stated	MTR	not provided	not provided	not stated	100	100	20	Cell culture medium + 1% FCS	Not given	Not given	Not given	not applicable	not given	not given	not given	not given	not given	not given	not given	not given	not given	Sphere	
EMTAB6396_A549, EMTAB6396_Beas2B, EMTAB6396_MRC9, EMTAB6396_THP1	Scala & Kinaret et al. 2018^83^	10.1016/j.impact.2018.05.003	Graphite nanofibers	GNF	Graphite nanofibers	Carbon	None	None	N/A	not stated	not stated	Sigma-Aldrich	not provided	not provided	not stated	10,000	140	32	Cell culture medium + 1% FCS	Not given	Not given	Not given	not stated	not given	not given	not given	not given	not given	not given	not given	not given	not given	Fiber	
EMTAB6396_A549, EMTAB6396_Beas2B, EMTAB6396_MRC9, EMTAB6396_THP1	Scala & Kinaret et al. 2018^83^	10.1016/j.impact.2018.05.003	Singlewalled carbon nanotube	SIG_SW	SWCNT (Sigma)	Carbon	None	None	N/A	not stated	not stated	Sigma-Aldrich	not provided	not provided	not stated	50,000	1.1	567	Cell culture medium + 1% FCS	Not given	Not given	Not given	not stated	not given	not given	not given	not given	not given	not given	not given	not given	not given	Tube	
EMTAB6396_A549, EMTAB6396_Beas2B, EMTAB6396_MRC9, EMTAB6396_THP1	Scala & Kinaret et al. 2018^83^	10.1016/j.impact.2018.05.003	Singlewalled carbon nanotube	SES_SW	SWCNT (SES)	Carbon	None	None	N/A	not stated	not stated	SES	not provided	not provided	not stated	1500	2	436	Cell culture medium + 1% FCS	Not given	Not given	Not given	not stated	not given	not given	not given	not given	not given	not given	not given	not given	not given	Tube	
EMTAB6396_A549, EMTAB6396_Beas2B, EMTAB6396_MRC9, EMTAB6396_THP1	Scala & Kinaret et al. 2018^83^	10.1016/j.impact.2018.05.003	Multiwalled carbon nanotube	BAY_MW	MWCNT (Bayer)	Carbon	None	None	N/A	not stated	not stated	Bayer	not provided	not provided	not stated	1000	14.5	204	Cell culture medium + 1% FCS	Not given	Not given	Not given	not stated	not given	not given	not given	not given	not given	not given	not given	not given	not given	Tube	
EMTAB6396_A549, EMTAB6396_Beas2B, EMTAB6396_MRC9, EMTAB6396_THP1	Scala & Kinaret et al. 2018^83^	10.1016/j.impact.2018.05.003	Multiwalled carbon nanotube	MIT_MW	MWCNT (Mitsui)	Carbon	None	None	N/A	not stated	not stated	Mitsui	not provided	not provided	not stated	13,000	50	22	Cell culture medium + 1% FCS	Not given	Not given	Not given	not stated	not given	not given	not given	not given	not given	not given	not given	not given	not given	Tube	
EMTAB6396_A549, EMTAB6396_Beas2B, EMTAB6396_MRC9, EMTAB6396_THP1	Scala & Kinaret et al. 2018^83^	10.1016/j.impact.2018.05.003	Multiwalled carbon nanotube	SES_MW	MWCNT (SES)	Carbon	None	None	N/A	not stated	not stated	SES	not provided	not provided	not stated	1500	20	60	Cell culture medium + 1% FCS	Not given	Not given	Not given	not stated	not given	not given	not given	not given	not given	not given	not given	not given	not given	Tube	
EMTAB6396_A549, EMTAB6396_Beas2B, EMTAB6396_MRC9, EMTAB6396_THP1	Scala & Kinaret et al. 2018^83^	10.1016/j.impact.2018.05.003	Multiwalled carbon nanotube	CHT_MW	MWCNT (Cheaptubes)	Carbon	None	None	N/A	not stated	not stated	cheaptubes	not provided	not provided	not stated	30,000	11.5	180	Cell culture medium + 1% FCS	Not given	Not given	Not given	not stated	not given	not given	not given	not given	not given	not given	not given	not given	not given	Tube	
EMTAB6396_A549, EMTAB6396_Beas2B, EMTAB6396_MRC9, EMTAB6396_THP1	Scala & Kinaret et al. 2018^83^	10.1016/j.impact.2018.05.003	Multiwalled carbon nanotube	SIG_MW	MWCNT (Sigma)	Carbon	None	None	N/A	not stated	not stated	Sigma-Aldrich	not provided	not provided	not stated	100,000	15	119	Cell culture medium + 1% FCS	Not given	Not given	Not given	not stated	not given	not given	not given	not given	not given	not given	not given	not given	not given	Tube	
GSE100500	Mohan et al. 2018^84^	10.1152/ajplung.00289.2017	Multi-walled CNTs	MWCNT	MWCNT	Carbon	None	None	N/A	99	<1% Fe	SES research	Houston, TX	900–1501	GS1801	20-40	20-40	not given			20–40		not stated	85.75	-46.3		850 ± 20 (160 ± 20 after centrifugation)			stable			https://www.sesres.com/carbon-nanotubes/	
GSE113088	Vis et al. 2018^86^	10.1021/acsnano.8b03363	Silica	USSN	nanoSiO2	Silica dioxide	None	None	N/A	not stated	not stated	In-house synthesized	Not applicable	Not applicable	Not applicable	3.6 ± 0.5	not applicable	not given																
GSE14452	Kawata et al. 2009^87^	10.1080/15287394.2016.1159635	Silver	Ag	AgNPs	Ag	polyethylenimine	None	N/A	not stated	not stated	Kyoto Nano Chemical Co., Ltd.	Kyoto, Japan	not provided	not provided	7-10	not applicable	not given	MEM	not provided	not provided	not provided	not provided	not provided	not provided	not provided	not provided	not provided	not provided	not provided	not provided	not provided	spherical	
GSE14452	Kawata et al. 2009^87^	10.1021/es900754q	PS	PS	PS	PS	None	None	N/A	not stated	not stated	Micromod	Germany	micromer^R^	not provided	15	not applicable	not given	MEM	not provided	not provided	not provided	not provided	not provided	not provided	not provided	not provided	not provided	not provided	not provided	not provided	not provided	spherical	
GSE14452	Kawata et al. 2009^87^	10.1021/es900754q	Ag_2_CO_3_	ionic control	Ag2CO3	None	None	None	N/A	not stated	not stated	Wako Pure Chemical Industries	Osaka, Japan	not provided	not provided	not provided	not applicable	not given	MEM	not provided	not provided	not provided	not provided	not provided	not provided	not provided	not provided	not provided	not provided	not provided	not provided	not provided	N/A	
GSE14452	Kawata et al. 2009^87^	10.1021/es900754q	Ag NPs + cysteine, a strong Ag^+^ ligand	N/A	AgNPs_cysteine	Ag	polyethylenimine	None	N/A	not stated	not stated	Kyoto Nano Chemical Co., Ltd.	Kyoto, Japan	not provided	not provided	7-10	not applicable	not given	MEM															
GSE17676	Peng et al. 2010^88^	10.1021/nl903043z	TiO2 nanotube array	TiO2-30	TiO2 nanotubes (30nm)	TiO2	None	None	anatase	>99%	not stated	In-house synthesized by anodic oxidation in an																						
electrolytic solution	Not applicable	Not applicable	not provided	30	1	not given	Surface array	not applicable	not applicable	not applicable	not applicable	not applicable	not applicable	not applicable	not applicable	not applicable	not applicable	not applicable	not applicable	not provided	vertical array													
GSE17676	Peng et al. 2010^88^	10.1021/nl903043z	TiO2 nanotube array	TiO2-100	TiO2 nanotubes (100nm)	TiO2	None	None	anatase	>99%	not stated	In-house synthesized by anodic oxidation in an																						
electrolytic solution	Not applicable	Not applicable	not provided	100	1	not given	Surface array	not applicable	not applicable	not applicable	not applicable	not applicable	not applicable	not applicable	not applicable	not applicable	not applicable	not applicable	not applicable	not provided	vertical array													
GSE19487_liver, GSE19487_lung	Halappanavar et al. 2011^89^	10.1002/em.20639	nanoTiO2	TiO2	nanoTiO2	TiO2	None	polyalcohol	rutile	not stated	zirconium, silicon, aluminum	Kemira	Pori, Finland	UV-titan L181	not provided	20	needle shaped	70						107.7	100 - 4,000									
GSE20692	Eom et al. 2014^90^	10.1016/j.toxlet.2014.05.019	Ag NPs	AgNPs	AgNPs	Ag	not stated	none	N/A	>99%	not stated	Sigma–Aldrich Chemical	St. Louis, MO, USA	not provided	<100	not applicable		dispersed in THF, then filtered through 100nm cellular acetate membrane	5−10	not applicable	not applicable	not given	not given	not given	28−35	not given		not given but < -30 mV	slightly agglomerated particles	not provided	spherical			
GSE27212	Fabbro et al. 2013^91^	10.1371/journal.pone.0073621	Multiwalled carbon nanotube	CNT	MWCNT	Carbon	pyrrolidine groups	None	N/A	>90%	<10% metal	Nanostructured & Amorphous Materials, Inc.	Katy, TX 77494, USA	not provided	not provided	20-30	not applicable	55	deposited on glass coverslip	not applicable	not applicable	not applicable	not applicable	not applicable	not applicable	not applicable	not applicable	not applicable	not applicable	not applicable	deposited on surface	not provided	Nanotube Conductive Scaffold	https://www.nanoamor.com/coa/coa_MWNT_1221YJ.pdf
GSE29110	Langley et al. 2011^92^	10.1080/15287394.2011.595669	SiO2	SiO2	Silica	None	None	N/A	N/A	99.20%	Al2O3 (0.03%), Fe2O3 (0.04%), TiO2 (0.02%)	U.S. Silica	Mill Creek, OK, USA	Min-U-Sil 5	not provided	1750 ± 50	not applicable	not given	aerosolized	not given	not given	not given	not given	not given	not given	not given	not given	not given	not given	not given	aersoloized	not provided	spherical	http://www.wrchem.com/wp-content/uploads/2015/11/MIN-U-Sil-5-Ground-Silica-TDS.pdf
GSE30178, GSE30180, GSE30213, GSE30214	Sellamuthu et al. 2011^65^	10.3109/08958378.2011.625995	Aerosolized silica	SiO2	Silica	None	None	N/A	N/A	99.20%	Al2O3 (0.03%), Fe2O3 (0.04%), TiO2 (0.02%)	US Silica	Berkeley Springs, WV, USA	Min-U-Sil 5	not provided	1750 ± 50	not applicable	not given	Sieved through a 10 μm mesh filter, serum-free F-12K		not provided	not applicable	not provided	not provided	not provided	not provided	not provided		not provided	Endotoxin-free	spherical			
GSE44294	Kodali et al. 2013^93^	10.1021/nn402145t	Superparamagnetic iron oxide (SPIO)	Fe3O4	SPIO	Fe3O4	None	None	N/A	not given	not given	Synthesis in-house	Not applicable	Not applicable	Not applicable	13	not applicable		RPMI	13	not applicable	not provided	not applicable	not provided	453	not provided	274	not provided	-33	-21.9	not provided	<0.01 EU/ml	spherical	
GSE44294	Kodali et al. 2013^93^	10.1021/nn402145t	Fluorescent-labeled amorphous silica nanospheres	SiO2	SiO2	SiO2	None	None	N/A	not given	not given	Corpuscular Inc.	Cold Spring, USA	not provided	not provided	50	not applicable		RPMI	50	not applicable	not provided	not applicable	not provided	87	not provided	87	not provided	-24	-23.5	not provided	<0.01 EU/ml	spherical	
GSE45598, GSE45868	Hussien et al. 2013^94^	10.1002/phy2.27	polymeric Eudragit RS NPs	Eudragit	Eudragit_RS	ethylacrylate and methylmethacrylate	quaternary ammonium groups	N/A	not given	not given	Evonik Industries	Essen, Germany	Eudragit RS 100 copolyme	Prepared in-house into NPs ((Eidi et al. 2010)	not applicable	not provided	not provided	not provided	not provided	not provided	not applicable	not provided	not provided	not provided	65.0 ± 26.3	not provided	+51.04		not provided	not provided	spherical			
GSE4567	Karoly et al. 2007^95^	10.1289/ehp.9556	Ambient ultrafine particles	UFP	UFP	Mixed	None	none	N/A	not given	Al, Cu, Fe, Pb, Si, Zn most abundant (Becker et al. 2005)	UFPs collected July 2002, Chapel Hill, North Carolina, outside US EPA health facility	not provided	not provided	not provided	not applicable	not provided	not provided	not provided	not provided	not provided	not applicable	not provided	not provided	not provided	not provided	not provided	not provided	not provided	not provided	not provided	spherical		
GSE48087	Chorley et al. 2014^96^	10.1016/j.neuro.2014.08.010	Ag NPs	Ag-10 cit	Ag10_Citrate	Ag	Citrate	none	N/A	>99.5	not stated	nanoComposix Inc.	San Diego, CA, USA	not provided	not provided	10	not applicable	53.7	0.2 mM citrate buffer	9.9 ± 1.9	Not appliable	Not appliable	not applicable	not given	7.5	low	not given	not given	not given	−10.28	monodispersed, monomodal	< 5	spherical	https://tools.nanocomposix.com:48/cdn/coa/Silver/Spheres/BioPure/AG10-SS-BP-CIT-HKE0021.pdf?107%20953
GSE48087	Chorley et al. 2014^96^	10.1016/j.neuro.2014.08.010	Ag NPs	Ag-10 PVP	Ag10_PVP	Ag	Citrate	PVP	N/A	>99.5	not stated	nanoComposix Inc.	San Diego, CA, USA	not provided	not provided	10	not applicable	53.5	0.2 mM citrate buffer	10.1 ± 1.8	Not appliable	Not appliable	not applicable	not given	60	low	not given	not given	not given	−7.25	monodispersed, monomodal	< 5	spherical	https://nanocomposix.com/collections/material-silver/products/biopure-silver-nanospheres-bare-citrate?variant=15906775597145#specs
GSE48087	Chorley et al. 2014^96^	10.1016/j.neuro.2014.08.010	Ag NPs	Ag-75 cit	Ag75_Citrate	Ag	None	PVP	N/A	>99.5	not stated	nanoComposix Inc.	San Diego, CA, USA	not provided	not provided	75	not applicable		HBSS		Not appliable	Not appliable	not applicable	not given	7.148	low	not given	not given	not given	−10.73	monodispersed, monomodal	< 2.5	spherical	https://tools.nanocomposix.com:48/cdn/coa/Silver/Spheres/BioPure/AG10-SS-BP-PVP-HKE0023.pdf?107%20953
GSE48087	Chorley et al. 2014^96^	10.1016/j.neuro.2014.08.010	Ag NPs	Ag-75 PVP	Ag75_PVP	Ag	none	PVP	N/A	>99.5	not stated	nanoComposix Inc.	San Diego, CA, USA	not provided	not provided	75	not applicable	7.5	HBSS	74.1 ± 8.2	Not appliable	Not appliable	not applicable	not given	70	low	not given	not given	not given	−6.27	monodispersed, monomodal	< 2.5	spherical	https://tools.nanocomposix.com:48/cdn/coa/Silver/Spheres/OECD/AG75-SS-BP-PVP-OECD-KJW1856.pdf?107%20953
GSE50176	Rydman et al. 2014^97^	10.1186/s12989-014-0048-2	long tangled MWCNT	tCNT	tCNT	Carbon	None	None	N/A	99.76 wt%	<1 wt% Ni and Fe	Cheap Tubes Inc.	Grafton, VT, USA	MWNTs 8-15 nm OD	not provided	8-15 (OD)	10-50	233	aerosolized						not provided	not provided	not provided	not provided	not provided	not provided	not provided	<0.022	long tubes	
GSE50176	Rydman et al. 2014^97^	10.1186/s12989-014-0048-2	long rigid MWCNT	rCNT	rCNT	Carbon	None	None	N/A	99.79 wt%	0.1 wt %	Mitsui & Co., Ltd.		XNRI MWNT-7	not provided	>50 (OD)	~13	26	aerosolized						not provided	not provided	not provided	not provided	not provided	not provided	not provided	<0.022	long tubes	
GSE51636	Chernova et al. 2017^98^	10.1016/j.cub.2017.09.007	Long Carbon Nanotubes	LNT	Long_carbon_nanotubes	Carbon	None	None	N/A	not stated	Fe (37.3), Cu (1.2), Ni (6.2) and Co (3.4)	University of Manchester, Produced and characterized by Dr. Ian Kinloch	Manchester, UK	not provided	not provided	20–100		20–1nh0en	DI water + 0.5%BSA	165.02 ± 4.68	not given	Not appliable	not applicable		not provided	not provided	not provided	not provided	not provided	not provided	not provided	<10	long tubes	
GSE51636	Chernova et al. 2017^98^	10.1016/j.cub.2017.09.007	South African amosite - long	LFA	Long_asbestos_fibres	Asbestos	None	None	amosite	not stated	Cd <0.1; Cr1.4; Co 3.4; Cu 5.2; Fe 853; Mn 104.8; Ni 5.1; Ti 2.0; V <0.1; Zn 27.3	Manville Corporation	USA	not provided	not provided	not stated	not stated	not stated	DI water + 0.5%BSA	1000	not given	Not appliable	not applicable		not provided	not provided	not provided	not provided	not provided	not provided	not provided	not provided	long tubes	
GSE51636	Chernova et al. 2017^98^	10.1016/j.cub.2017.09.007	South African amosite - short	SFA	Short_asbestos_fibres	Asbestos	None	None	amosite	not stated	Cd <0.1; Co 2.1; Cr <0.1; Cu 3.1; Fe 547; Mn 36.3; Ni 18.4; Ti 31.5; Vn 3.1; Zn 10.5	Manville Corporation	USA	not provided	not provided	not stated	not stated	not stated	DI water + 0.5%BSA	700	not given	Not appliable	not applicable		not provided	not provided	not provided	not provided	not provided	not provided	not provided	not provided	long tubes	
GSE51636	Chernova et al. 2017^98^	10.1016/j.cub.2017.09.007	Short Carbon Nanotubes	SNT	Short_carbon_nanotubes	Carbon	None	None	N/A	not stated	Cd <0.1; Cr <0.1; Co <0.1; Cu <0.1; Fe 24.2; Mn 50.3; Ni 21.6; Ti 0.4; V <0.1; Zn 5.3	Nanostructured & Amorphous Materials	TX, USA	#1246YJS	not provided	not stated	not stated	not stated	DI water + 0.5%BSA	125	not given	Not appliable	not applicable		not provided	not provided	not provided	not provided	not provided	not provided	not provided	not provided	long tubes	
GSE51661	Aday et al. 2014^75^	10.1039/C4RA04041D	Poly(lactic acid-co-glycolic acid)	NP210-PFCE	PLGA	PLGA	protamine sulfate (cationic)	None	N/A	not stated	not stated	In-house synthesized	Not applicable	Not applicable	Not applicable	not givem	not applicable	not given	M200 or QBSF-60	not provided	not provided	not provided	not provided	not provided	210	0.12 + 0.01	not provided	not provided	7.0 ± 1.7	not provided	not provided	not given	spherical	
GSE7010	Huang et al. 2011^99^	10.1080/15287394.2010.516238	Particulate matter	PM coarse	Chapel_hill_coarse	Nitrate & metals	None	None	crustal material (Al, Fe, and Si)	not stated	Collected Oct 2010, Chapel Hill, NC, outside the U.S. EPA	not provided	2500-10000	not applicable	2500ru0etg	sterile distilled water	not provided	not provided	not provided	not provided	not provided	not provided	not provided	not provided	not provided	not provided	not provided	not provided	not provided	roughly spherical				
GSE7010	Huang et al. 2011^99^	10.1080/15287394.2010.516238	Particulate matter	PM fine	Chapel_hill_fine	Nitrate & metals	None	None	N/A	not stated	As, Pb, V, Zn, Br, EC	Collected Oct 2010, Chapel Hill, NC, outside the U.S. EPA	not provided	100-2500	not applicable	100-ru0etg	sterile distilled water	not provided	not provided	not provided	not provided	not provided	not provided	not provided	not provided	not provided	not provided	not provided	not provided	not provided	roughly spherical			
GSE7010	Huang et al. 2011^99^	10.1080/15287394.2010.516238	Particulate matter	PM ultrafine	Chapel_hill_UFP	Nitrate & metals	None	None	N/A	not stated	As, Pb, V, Zn, Br, EC	Collected Oct 2010, Chapel Hill, NC, outside the U.S. EPA	not provided	<100	not applicable	not given	sterile distilled water	not provided	not provided	not provided	not provided	not provided	not provided	not provided	not provided	not provided	not provided	not provided	not provided	not provided	roughly spherical			
GSE79766	Gao et al. 2017^100^	10.1186/s12951-017-0265-6	Silver NPs	BioPure Ag-20	AgNPs	Ag	citrate	None	N/A	99.99	not stated	Nanocomposix	San Diego, CA	not provided	not provided	20	not applicable	not given	ESGRO Complete Basal Medium	20.4 ± 3.2	not applicable	not applicable	not applicable	not given	20.4 ± 3.2	0.29	78.6	0.048	−39.8 (2mM citrate)	not given	monodispersed, monomodal	<5	spherical	https://tools.nanocomposix.com:48/cdn/coa/Silver/Spheres/BioPure/AG20-SS-BP-CIT-SCM0069.pdf?107%20953
GSE82062	Guo et al. 2017^101^	10.1080/17435390.2017.1403658	Amorphous SiO_2_	Silica	nanoSiO2	SiO_2_	None	None	N/A	>99.9%	not stated	In-house synthesis using Stober method	not applicable	not applicable	Not applicable	60	not applicable	not given	DMEM	57.66 ± 7.30	not applicable	not applicable	not applicable	not given	not given	not given	106	not given	not given	−32	monodispersed, monomodal	not given	spherical	not applicable
GSE85711	Kinaret et al. 2017^102^	10.1021/acsnano.6b05652	long tangled MWCNT	tCNT	tCNT	Carbon	None	None	N/A	99.76 wt%	<1 wt% Ni and Fe	Cheap Tubes Inc.	Grafton, VT, USA	MWNTs 8-15 nm OD	not provided	8-15 (OD)	10-50	233	aerosolized													<0.022	long tubes	
GSE85711	Kinaret et al. 2017^102^	10.1021/acsnano.6b05652	long rigid MWCNT	rCNT	rCNT	Carbon	None	None	N/A	99.79 wt%	0.1 wt %	Mitsui & Co., Ltd.		XNRI MWNT-7	not provided	>50 (OD)	~13	26	aerosolized													<0.022	long tubes	
GSE92563	Proquin et al. 2018^103^	10.1016/j.dib.2017.11.067	Titanium dioxide	E171	TiO2 (E171)	TiO_2_	None	None	anatase	99%	Al2O3 and/or SiO2	Sensient Technologies Company	Mexico	not given	not given	120	not applicable	not applicable	water	not given	not applicable	not applicable	not applicable	not given	not given	not given	316.8 ± 282.4	not given	not given	−12.78 ± 0.52	E171 comprises 2 fractions : 39% NPs and 61% MPs	rounded / spherical	Couldn’t find the specific one used	
GSE92900	Kinaret et al. 2017^81^	10.1021/acsnano.6b08650	long rigid MWCNT	rCNT	rCNT	Carbon	None	None	N/A	99.79 wt%	not stated	Mitsui & Co.	Tokyo, Japan	MWCNT-7	Not given			26	PBS with 0.6 mg of BSA/mL	N/A	50	13000	not applicable	22	not given	not given	not given	not given	not given	not given	not given	Not given	tube	
GSE92900	Kinaret et al. 2017^81^	10.1021/acsnano.6b08650	long tangled MWCNT	tCNT	tCNT	Carbon	None	None	N/A	>95	not stated	Cheaptubes Inc.	Grafton, VT, USA	Cheaptubes	Not given	8-15	10-50	233	PBS with 0.6 mg of BSA/mL	N/A	11.5	30000	not stated	180	not given	not given	not given	not given	not given	not given	not given	not stated	tube	https://www.cheaptubes.com/product/multi-walled-carbon-nanotubes-8-15nm/
GSE92900	Kinaret et al. 2017^81^	10.1021/acsnano.6b08650	short tangled MWCNT	Baytube	MWCNT (Baytube)	Carbon	None	None	N/A	99	not stated	Bayer Material Science	Laufenburg, Germany	Baytubes C150 HP	Not given	5-20	1-10	not given	PBS with 0.6 mg of BSA/mL	N/A	14.5	1000	not stated	204	not given	not given	not given	not given	not given	not given	not given	not stated	tube	Not produced anymore - no specification available
GSE92900	Kinaret et al. 2017^81^	10.1021/acsnano.6b08650	long rigid carbon fiber graphite	graphite	Graphite	Carbon	None	None	N/A	not stated	not stated	Sigma-Aldrich	Finland	636398	Not given	not given	not given	not given	PBS with 0.6 mg of BSA/mL	N/A	140	10000	not applicable	32	not given	not given	not given	not given	not given	not given	not given	Not given	fibre	
GSE92900	Kinaret et al. 2017^81^	10.1021/acsnano.6b08650	hollow carbon sphere	fullerene	Fullerene	Carbon	None	None	N/A	not stated	not stated	MTR Ltd.	Cleaveland, OH, USA	MTS60	Not given	1	not applicable	not stated	PBS with 0.6 mg of BSA/mL	N/A	100	100	not applicable	20	not given	not given	not given	not given	not given	not given	not given	Not given	sphere	
GSE92900	Kinaret et al. 2017^81^	10.1021/acsnano.6b08650	short rigid MWCNT	SES	MWCNT (SES)	Carbon	None	None	N/A	>95%	not stated	SES Research	Houston, Texas	900−1260	Not given	10-30	1-2		PBS with 0.6 mg of BSA/mL	N/A	20	1500	not applicable	60	not given	not given	not given	not given	not given	not given	not given	Not given	tube	https://www.sesres.com/carbon-nanotubes/
GSE92987	Kalmodia et al. 2017^104^	10.1016/j.omtn.2017.10.012	Functionalised gold NPs	GNPs	AuNPs	Gold	citrate	none	N/A	not given	not given	In-house synthesis using extracts of *Vitis vinefera L*.	not applicable	not applicable	not applicable	not provided	not applicable	not provided	water	Not given	Not applicable	Not applicable	Not applicable	not given	not given	not given	24.47 ± 0.028	0.388	not given	-18.2	monodispersed, monomodal	not given	spherical	not applicable
GSE92987	Kalmodia et al. 2017^104^	10.1016/j.omtn.2017.10.012	Functionalised gold NPs	GNPs	AuNPs-HDM2	Gold	none	HDM2	N/A	not given	not given	In-house synthesis using extracts of *Vitis vinefera L*.	not applicable	not applicable	not applicable	not provided	not applicable	not provided	water	Not given	Not applicable	Not applicable	Not applicable	not given	not given	not given	66.72 ± 2.83	0.303	not given	-11.4	monodispersed, monomodal	not given	spherical	not applicable
GSE96720	Li et al. 2018^105^	10.1093/toxsci/kfy090	Functionalised Graphene oxide	OH-GQDs	Graphene quantum dots (GQD)	Carbon	hydroxyl	none	N/A	not given	not given	XFNANO Materials Tech Co.	Nanjing, China	XF091	not provided	4-6	not applicable	not given	RPMI 1640 or DMEM	4-6	Not applicable	not applicable	Not applicable	not given	14.1 ± 2.3	not given	not given	not given	not given	not given	monodispersed, monomodal	not given	spheres	https://www.xfnano.com/HaodeUpload/productfile/201904/20190417/XF091-%E7%BE%9F%E5%9F%BA%E5%8C%96%E7%9F%B3%E5%A2%A8%E7%83%AF%E9%87%8F%E5%AD%90%E7%82%B9%20-%20%E7%AC%AC%E4%BA%8C%E6%AC%A1%E4%BF%AE%E8%AE%A220180925.pdf
GSE99929_Jurkat, GSE99929_THP1	Orecchioni et al. 2017^106^	10.1038/s41467-017-01015-3	Graphene oxide	GO	GO	Carbon	none	none	N/A	not stated	not stated	In-house produced from graphite flakes (Barnwell, UK) using modified Hummers’ method (Ali-Boucetta et al. 2013)	not applicable	not applicable	not applicable	<300	<300	not given	RPMI-1640 with FBS 10%	1	245.7 ± 57.1	N/A	2-3 layers	not given	not applicable	not applicable	not applicable	not applicable	not applicable	not applicable	not described	not given	sheets	not applicable
GSE99929_Jurkat, GSE99929_THP1	Orecchioni et al. 2017^106^	10.1038/s41467-017-01015-3	Amine-modified Graphene oxide	GONH_2_	GONH2	Carbon	triethyleneglycol (TEG) diamine	none	N/A	not stated	not stated	In-house produced from graphite flakes (Barnwell, UK) using modified Hummers’ method (Ali-Boucetta et al. 2013)	not applicable	not applicable	not applicable	<300	<300	not given	RPMI-1640 with FBS 10%	1	184.4 ± 115.4	N/A	2-3 layers	not given	not applicable	not applicable	not applicable	not applicable	not applicable	not applicable	not described	not given	sheets	not applicable
GSE101992	Yazdimamaghani et al. 2020^109^	10.1016/j.nano.2017.11.021	Silica dioxide	Stöber 50 a	Silica NPs (Stober50)	SiO_2_	none	none	N/A	not stated	not stated	In-house prodced using a modified Stober method	N/A	N/A	N/A	46	N/A	N/A	RPMI + 10% FBS	46 ± 4.9	N/A	N/A	N/A	186.03	108.1 ± 6.3	not given	190.5 ± 4.8	not given	−40.3 ± 1.9	−9.57 ± 0.4	monodispersed, monomodal	not given	spherical	not applicable
GSE101992	Yazdimamaghani et al. 2020^109^	10.1016/j.nano.2017.11.021	Silica dioxide	Stöber 500 a	Silica NPs (Stober500)	SiO_2_	none	none	N/A	not stated	not stated	In-house prodced using a modified Stober method	N/A	N/A	N/A	436	N/A	N/A	RPMI + 10% FBS	432 ± 18.7	N/A	N/A	N/A	6.83	520.3 ± 4.8	not given	539.7 ± 6.2	not given	−53.1 ± 1.5	−9.48 ± 0.5	monodispersed, monomodal	not given	spherical	not applicable
GSE101992	Yazdimamaghani et al. 2020^109^	10.1016/j.nano.2017.11.021	Silica dioxide	MSN500 a	Silica NPs (MSN500)	SiO_2_	none	none	mesoporous	not stated	not stated	In-house prodced using a modified Stober method	N/A	N/A	N/A	462	N/A	N/A	RPMI + 10% FBS	466 ± 86	N/A	N/A	N/A	950.09	799.8 ± 22.9	not given	915 ± 72	not given	−44 ± 0.8	−8.03 ± 0.5	monodispersed, monomodal	not given	spherical	not applicable
GSE101992	Yazdimamaghani et al. 2020^109^	10.1016/j.nano.2017.11.021	Silica dioxide	MSN500-PEG	Silica NPs (MSN_PEG500)	SiO_2_	none	PEG (10kD)	mesoporous	not stated	not stated	In-house prodced using a modified Stober method	N/A	N/A	N/A	462	N/A	N/A	RPMI + 10% FBS	466 ± 86	N/A	N/A	N/A	526.29	706.5 ± 22.2	not given	757 ± 62	not given	−34.3 ± 0.7	−8.56 ± 0.8	monodispersed, monomodal	not given	spherical	not applicable
GSE125742_HBMSC, GSE125742_HUVEC, GSE125742_mouse_bone, GSE125742_mouse_heart, GSE125742_mouse_kidney, GSE125742_mouse_liver, GSE125742_mouse_muscle, GSE125742_mouse_MuscleStemCell, GSE125742_mouse_PBMC, GSE125742_mouse_spleen	Wu et al. 2019^110^	10.1016/j.biomaterials.2019.03.019	Hydroxyapatite	HANP	Hydroxyapatite	Ca_10_(PO_4_)_6_(OH)_2_	none	none	N/A	not stated	not stated	Ji’nan Daigang Biomaterial Co. Ltd.	Shandong, China	not given	not given	20	80	not given	saline (0.9% sodium chloride)	N/A	20	80	N/A	not provided	not provided	not provided	100	not provided	not provided	not provided	partially agglometated in medium	not given	rod-shaped	not available
GSE143717	House et al. 2020^108^	10.1002/smll.202000299	AgNPs	Ag-cit	AgNPs_Citrate	Ag	citrate	None	N/A	99.90%	not stated	nanoComposix	San Diego, CA, USA	WJT0044	not given	40	N/A	14.2	DMEM	41 ± 5	N/A	N/A	N/A	not provided	42	not provided	96.75±5.48	not provided	-49	not provided	monodispersed, monomodal	< 2.5	spherical	https://tools.nanocomposix.com:48/cdn/coa/Silver/Spheres/BioPure/AG40-SS-BP-CIT-JEA0271.pdf?107%20953
GSE143717	House et al. 2020^108^	10.1002/smll.202000299	AgNPs	Ag-PEG	AgNPs_polyethylene_glycol	Ag	none	PEG	N/A	99.90%	not stated	nanoComposix	San Diego, CA, USA	AGGB40	CWW0059	40	N/A	13.8	DMEM	39 ± 4	N/A	N/A	N/A	not provided	60	not provided	71.00±1.97	not provided	-29	not provided	monodispersed, monomodal	< 2.5	spherical	https://tools.nanocomposix.com:48/cdn/coa/Silver/Spheres/BioPure/AG40-SS-BP-PEG-JCP1525.pdf?107%20953
GSE143717	House et al. 2020^108^	10.1002/smll.202000299	AgNPs	Ag-bPEI	AgNPs_branched_polyethylenimine	Ag	none	branched PEI	N/A	99.90%	not stated	nanoComposix	San Diego, CA, USA	AGGB40	PSK0027	40	N/A	14.4	DMEM	39 ± 3	N/A	N/A	N/A	not provided	65	not provided	149.14±15.95	not provided	63.5	not provided	monodispersed, monomodal	< 2.5	spherical	https://tools.nanocomposix.com:48/cdn/coa/Silver/Spheres/BioPure/AG40-SS-BP-BPEI-SCM0040.pdf?107%20953
GSE153419	Yang & Landry 2020^111^	10.1101/2020.06.30.181420	Short SWCNTs	(GT)6 SWCNT	(GT)6 SWCNT	Carbon	none	(GT)6 DNA	N/A	not given	not given	NanoIntegris	Quebec, Canada	HiPco™SWCNTs	not given	~0.8 – 1.2	~100 – 1000	~400 – 1000	DMEM/F12 + 10% FBS	not given	not given	not given	1	not given	not given	not given	not given	not given	not given	not given	not given	Below detection limit	short tubes	http://nanointegris.com/our-products/small-diameter-swnts-hipco/
GSE153419	Yang & Landry 2020^111^	10.1101/2020.06.30.181420	SWCNTs	SWCNT-COOH	Carboxylated SWCNT	Carbon	COOH	None	N/A	>90%	5-8% metals	Sigma Aldrich	USA	COOH-SWCNTs	not given	4-5	0.5-1.5		DMEM/F12 + 10% FBS	not given	not given	not given	1	not given	not given	not given	not given	not given	not given	not given	not given	Below detection limit	short tubes	https://api.sigmaaldrich.com/deepweb/assets/sigmaaldrich/quality/spec/401/763/652490-BULK_______ALDRICH__.pdf
GSE155027	Kang et al. 2020^107^	10.1038/s41598-020-70415-1	Ag_2_S NPs	Ag_2_S	Ag2S	Ag	none	none	monoclinic	not stated	not stated	In-house synthesized	N/A	N/A	N/A	N/A	N/A	N/A	DMEM + 10% FBS	<10	N/A	N/A	N/A	N/A	25.67	0.56	not given	not given	31.73	not given	not given	not given	spherical	not applicable
GSE155027	Kang et al. 2020^107^	10.1038/s41598-020-70415-1	Li-doped Ag_2_S NPs	Li-doped Ag_2_S	Li-doped Ag2S	Ag	none	none	monoclinic	not stated	not stated	In-house synthesized	N/A	N/A	N/A	N/A	N/A	N/A	DMEM + 10% FBS	<10	N/A	N/A	N/A	N/A	53.08	0.379	not given	not given	26.38	not given	not given	not given	spherical	not applicable
GSE86339_C57Bl6, GSE86339_DBA2	Frank et al. 2017^112^	10.1016/j.taap.2017.04.019	Multi-walled CNTs	MWCNT	MWCNT	Carbon	none	none	N/A	99	negligible metal content	Baytubes	Leverkusen, German	not given	not given	N/A	10.7	>1	PBS with 1% Pluronic F127	N/A	10.7±3.1	>1	N/A	193.6	not given	not given	not given	not given	not given	not given	loose agglomerates of fibers	Below detection limit	tangled morphology	not available
