# Supplementary material for: Manually curated transcriptomics data collection for toxicogenomic assessment of engineered nanomaterials
Source: Sci Data. 2021 Feb 8;8:49. doi: 10.1038/s41597-021-00808-y (PMC7870661; doi:10.1038/s41597-021-00808-y)
Supplement: Supplementary file 1 [file 41597_2021_808_MOESM1_ESM.pdf]

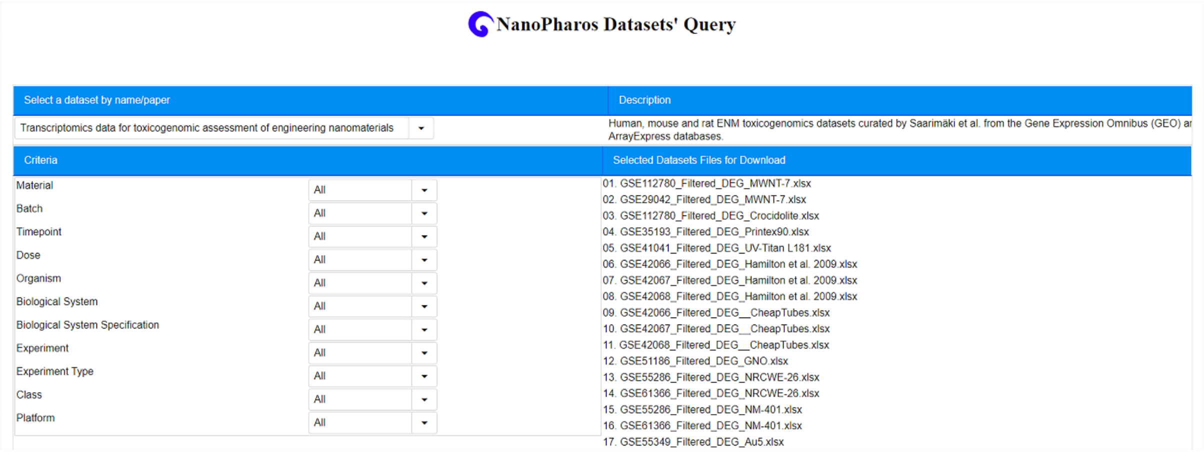

Supplementary File 1. The NanoPharos database provides users with free access to ready-for-modelling datasets. The user can filter the available toxicogenomics datasets based on a number of options to meet specific requirements.
